# Targeting oncogenic TβRI signaling inhibits androgen-independent prostate cancer growth and metastasis

**DOI:** 10.1038/s41392-026-02737-x

**Published:** 2026-06-17

**Authors:** Per Flodbring Larsson, Alexej Schmidt, Yabing Mu, Guangxiang Zang, Jie Song, Vishnupriya Gajavilli, Junting Tao, Olena Rakhimova, Madelene Ericsson, Karthik Aripaka, Sofia Halin Bergström, Wei Yuan, Denisa Bogdan, Aaron Huairen Zhang, Jon Welti, Anders Bergh, Johann de Bono, Carl-Henrik Heldin, Maréne Landström

**Affiliations:** 1 Department of Medical Bioscience, Building 6 M, Umeå University, Umeå, Sweden; 2 The Institute of Cancer Research and Royal Marsden Hospital, London, UK; 3 Department of Medical Biochemistry and Microbiology, SciLifeLab, Uppsala University, Uppsala, Sweden

**Keywords:** Drug development, Urological cancer

## Abstract

Metastatic castration-resistant prostate cancer (mCRPC) remains the primary cause of prostate cancer-related mortality. Despite the availability of treatments, the molecular mechanisms underlying tumor invasion and metastasis are not fully understood, highlighting the need for novel therapeutic strategies. In this study, we developed fully human monoclonal antibodies (mAbs) that prevent the proteolytic cleavage of the transforming growth factor-beta (TGFβ) type I receptor (TβRI) by steric hindrance. This cleavage, mediated by the metalloprotease ADAM17 (a disintegrin and metalloprotease domain 17; also known as TACE), results in the generation of a soluble intracellular domain (TβRI-ICD) that is translocated to the nucleus of castration-resistant prostate cancer (CRPC) cells and promotes epithelial-to-mesenchymal transition (EMT), invasion, and metastasis. High levels of *TGFBR1* correlated with poor survival in two independent clinical cohorts of patients with mCRPC, and a strong positive correlation between *TGFBR1* and *ADAM17* expression was observed. In a preclinical human orthotopic mCRPC mouse model, treatment with therapeutic mAbs effectively prevented the nuclear accumulation of TβRI-ICD, inhibited EMT, and suppressed tumor growth, invasion, and metastasis. Notably, the therapeutic effect was comparable to that of docetaxel, a current standard-of-care chemotherapy, without noticeable side effects on body weight, proximal aorta or heart function detected in immune-deficient mice. These findings suggest that targeting TβRI cleavage using specific mAbs is a novel precision medicine approach for the treatment of mCRPC. By selectively blocking the prometastatic activity of TβRI-ICD without disrupting physiological TGFβ signaling, this strategy may provide a safer and more effective alternative to existing therapies for advanced prostate cancer.

## Introduction

Cancer is the second most common cause of death worldwide, accounting for approximately 9.8 million deaths in 2020,^1^ approximately 90% of which are due to metastases. Prostate cancer is the second most deadly form of cancer in men,^2,3^ and androgen receptor (AR) signaling plays a crucial role in prostate cancer initiation and disease progression.^4^ Initially, patients are treated with androgen withdrawal;^5^ however, when the cancer no longer responds to this treatment, the tumor becomes castration resistant.^4^ Patients with metastatic castration-resistant prostate cancer (mCRPC) have an unfavorable prognosis, as effective treatment options are still lacking.^6^

Transforming growth factor β (TGFβ) plays important roles in the growth and metastasis of prostate cancer, breast carcinoma and many other tumors.^7^ Thus, the development of therapeutic strategies targeting TGFβ signaling has been pursued and has entered clinical trials; however, the pleiotropic physiological role of TGFβ has limited its clinical usefulness,^8^ and thus so far, no treatment strategies targeting TGFβ in cancer have been approved for clinical use.^9^ High levels of TGFβ have been detected in the blood of patients with prostate cancer and with invasive disease.^10^ TGFβ transduces its effects through transmembrane TGFβ Type I and Type II receptors (TβRI and TβRII, respectively). Activated TβRI transduces its effects via the canonical SMAD signaling pathway through the phosphorylation of SMAD2 and SMAD3 and by the activation of noncanonical signaling pathways.^7,11–13^ Physiological TGFβ-SMAD signaling is necessary for the maintenance of cellular homeostasis; for the differentiation of epithelial cells, cardiomyocytes and cells in the proximal aorta; and for the regulation of the immune response.^7,14^ In cancer cells, TGFβ plays an ambiguous role, first being a tumor suppressor, by inhibiting the growth of most cell types and inducing apoptosis. However, in advanced cancers, TGFβ promotes epithelial to mesenchymal transition (EMT), tumor cell invasion and metastasis, as well as angiogenesis and the development of cancer-associated fibroblasts, while it dampens the immune response against cancer cells.^7,9,12,15,16^ TGFβ promotes prostate cancer progression, and TβRI expression is increased in aggressive forms of prostate cancer.^17^ The protumorigenic effects of TβRI were further demonstrated using a preclinical orthotopic model. CRISPR/Cas9-mediated knockout of TβRI in a human metastatic castration-resistant prostate cancer (mCRPC; PC-3U) cell line resulted in a significant reduction in primary tumor growth as well as the metastatic burden to regional lymph nodes in vivo.^18^ Moreover, the expression of *TGFBR1* is increased in many different forms of cancer.^19^ Thus, specific reduction of the expression of *TGFBRI* in cancer cells may hinder tumor growth and metastasis of advanced cancers.

Owing to the well-established tumor-promoting effects of TGFβ, attempts to target TGFβ have been made and are currently being investigated in clinical trials.^14^ Previous efforts to block TGFβ signaling in cancer cells by small molecules that inhibit the kinase activity of TβRI have been reported to lead to unwanted side effects on the heart and proximal aorta because of the important physiological effects of TGFβ in these organs.^8^ Therefore, it would be beneficial to specifically inhibit the tumor-promoting effects of TGFβ in patients with prostate cancer without interfering with physiological systemic effects of TGFβ.

Transmembrane TβRIs undergo proteolytic cleavage in cancer cells by ADAM17 (a disintegrin and metalloprotease domain 17), also called tumor necrosis factor-α-converting enzyme (TACE), followed by proteolytic cleavage of the transmembrane domain by γ-secretase.^16,20,21^ The intracellular domain (TβRI-ICD) is subsequently transported to the nucleus, where it binds the transcriptional coactivator p300 and promotes the expression of *SNAI1*, a gene implicated in EMT and invasion of cancer cells.^21–23^ Our previous investigations utilizing chromatin immunoprecipitation demonstrated that nuclear TβRI-ICD binds to its own promoter in TGFβ-stimulated mCRPC (PC-3U) cells, resulting in upregulation of its expression.^22^ This evidence indicates that the oncogenic TβRI-ICD pathway promotes sustained TGFβ signaling in a positive feedback manner and is thereby contributing to tumor progression. Furthermore, recent findings revealed that TβRI expression in mCRPC cells is essential for the induction of secreted thrombospondin 1 (THBS1),^18^ a multifunctional glycoprotein integral to the extracellular matrix. Notably, THBS1 has been shown to activate latent TGFβ^24,25^ which amplifies tumor progression through enhanced TGFβ signaling. Experimental evidence has also demonstrated that THBS1 is required for both migration and invasion of mCRPC cells. Mechanistically, it was reported that TβRI interacts with THBS1 and the heterodimeric transmembrane adhesion receptor integrin αV (ITGAV) in the leading edge of the cell during cell migration.^18^

Here, we present a novel treatment strategy aimed at preventing the formation of TβRI-ICDs. We show that an affinity-matured, fully human monoclonal antibody (mAbF11) prevents the generation of TβRI-ICD and its subsequent nuclear accumulation without interfering with physiological TGFβ signaling via the canonical SMAD pathway. Treatment with this antibody prevents the nuclear TβRI-ICD-p300 complex, the expression of EMT-related genes, the invasion of mCRPC cells in vitro, and their growth and metastases to regional lymph nodes in vivo in a preclinical mCRPC model.

## Results

### Targeting TβRI-ICDs prevents tumor growth and metastasis in castration-resistant prostate cancer

**Fig. 1:**
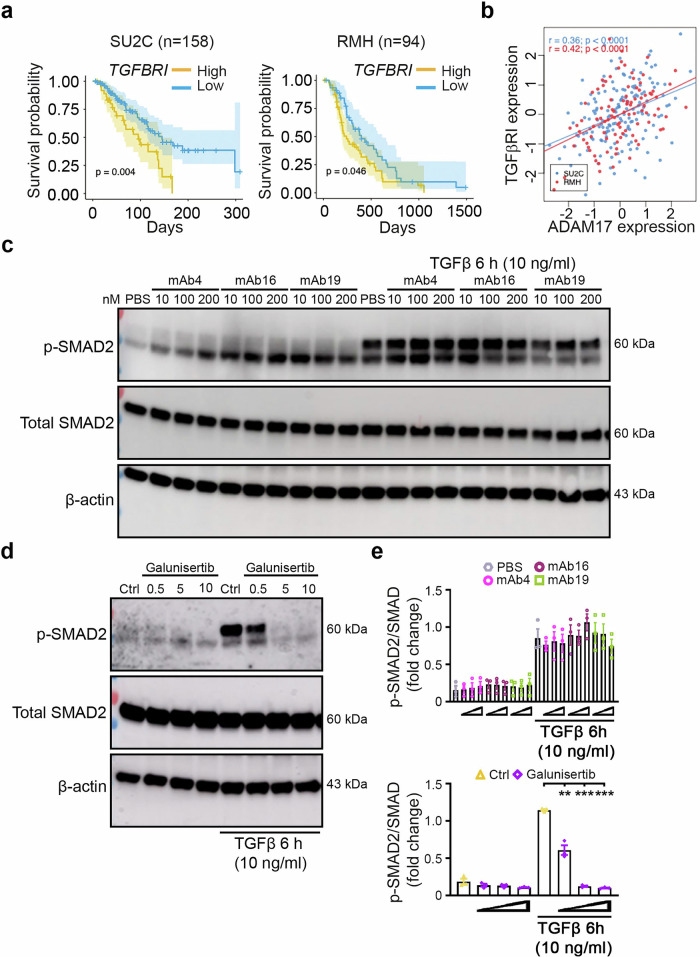
TGFβRI is a potential target in metastatic castration-resistant prostate cancer. **a** Analyses of the correlation between *TGFBR1* expression and survival (**b**) and between *TGFBR1* expression and *ADAM17* expression in the SUC2C and RMH databases. **c**, **d** TβRI-HA–reconstituted A9/PC-3U cells were pretreated with increasing concentrations of antibodies (10–200 nM), PBS, or galunisertib (0.5–10 µM) or vehicle (0.1% DMSO) and subsequently stimulated with or without TGF-β1 for 6 hours. or (**d**) increasing concentration of the TβRI kinase inhibitor galunisertib (0.5 μM, 5 μM, 10 μM) or vehicle (Control/Ctrl; i.e., DMSO). The cell lysates were subjected to immunoblotting with antibodies against pSMAD2, SMAD2, and β-actin. Images from representative immunoblot experiments. **e** The intensity of immunoblots in (**c**, **d**) was measured, and the ratio of pSMAD2/total SMAD2 is depicted as the normalized signal of pSMAD2. The data are presented as the mean values ± SEMs from three independent experiments. Statistical significance was determined by Student’s *t* test: **P < 0.01; ***P < 0.001

**Fig. 2:**
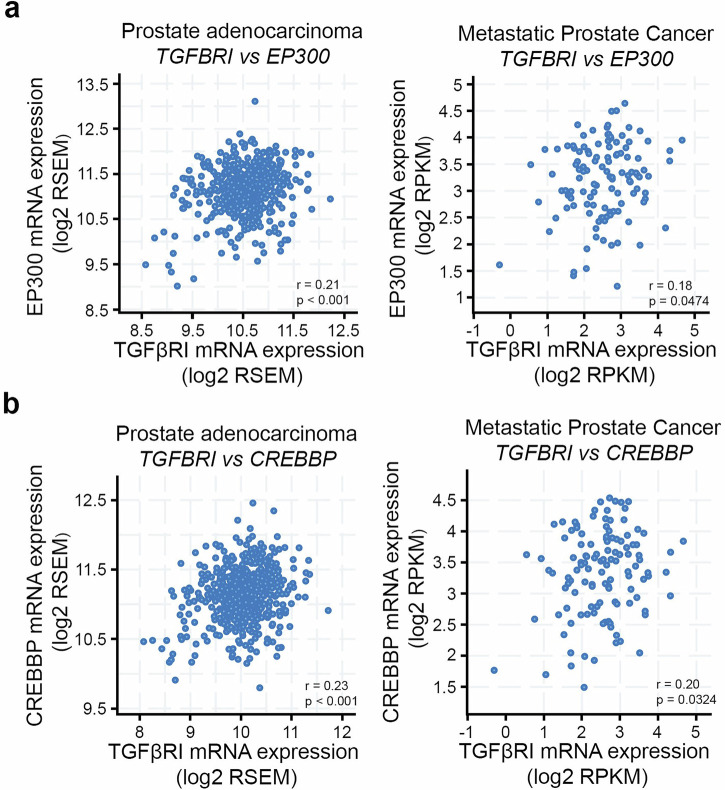
Correlation between *TGFBR1* and *EP300/CREBBP* expression in prostate adenocarcinoma and metastatic prostate cancer. **a** Scatter plot showing Pearson correlation between mRNA expression of *TGFBR1* and *EP300* encoding p300 protein (log2), in the TCGA prostate adenocarcinoma (left) and SU2C metastatic prostate cancer (right) cohorts described by Robinson et al. ^30^. **b** Scatter plots showing the Pearson correlation between mRNA expression of *TGFBR1* and *CREBBP* encoding CBP (log2), in the TCGA prostate adenocarcinoma (left) and SU2C metastatic prostate cancer (right) cohorts

**Fig. 3:**
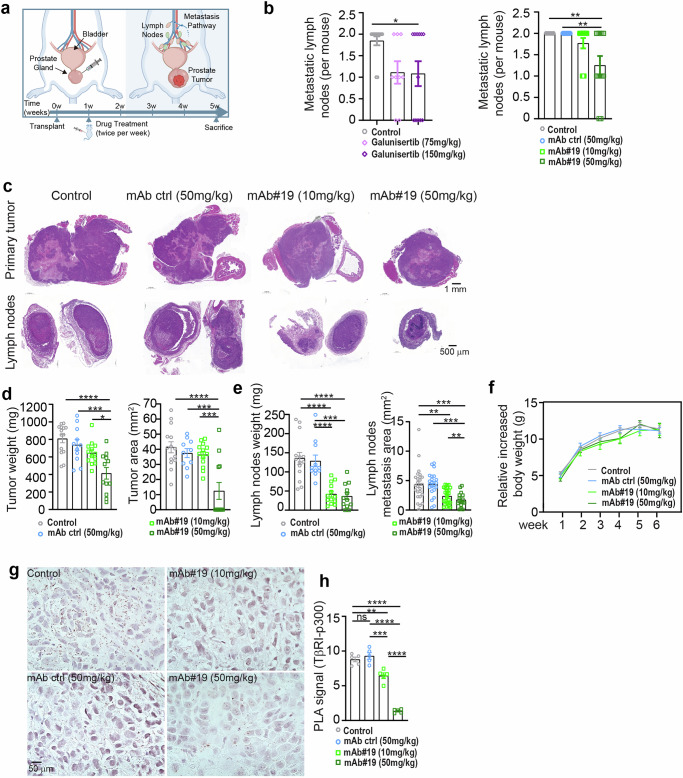
Effects of galunisertib and mAb19 in a murine orthotopic metastatic castration-resistant prostate cancer model. **a** Illustration of the murine orthotopic xenograft metastatic castration-resistant prostate cancer (mCRPC) model and metastases to regional lymph nodes and a timeline of the in vivo experiment. Figure was created by Biorender. **b** In vivo evaluation of the efficacy of treatment with a TβRI kinase inhibitor galunisertib or a monoclonal antibody (mAb), an isotype-specific control mAb (control), or mAb19 at the indicated doses in the mCRPC model. **c** H&E staining of primary tumor tissue and metastatic lesions in regional lymph nodes showing the morphology of the tissues. Scale bar, 1 mm for the primary tumor and 500 μm for the lymph nodes. **d** Tumor weight and cross-sectional area (measured as the largest diameter in H&E-stained sections). **e** Lymph node weight and metastatic burden quantification were performed by measuring the metastatic area in the lymph node. **f** Relative increase in body weight of mice monitored weekly over six weeks. **g** In situ proximity ligation assay (PLA) of TβRI and p300 in tumor tissues to assess the inhibitory effect of mAb19 on the nuclear TβRI-ICD—p300 complex in vivo. Scale bar, 50 μm. **h** Quantification of the PLA signal. The data are presented as the means ± SEMs. Statistical significance was determined using one-way ANOVA with Tukey’s multiple comparisons test or Kruskal–Walli’s test for non-parametric data. *P < 0.05, **P <0.01, ***P < 0.001, ****P < 0.0001, ns not significant

In previous studies, we demonstrated that nuclear TβRI-ICD binds to p300, promoting TβRI expression and the invasion of androgen-independent prostate cancer PC-3U cells.^20,21^ Preventing its proteolytic cleavage could represent a novel therapeutic strategy to hinder tumor growth and metastasis in mCRPC. To further study the role of TβRI in mCRPC, we analyzed the expression of *TGFBR1* in two different clinical cohorts of mCRPC clinical biopsy RNAseq data, SU2C^26^ (*n* = 158) and RMH^27^ (*n* = 94), and found that high levels of *TGFBR1* correlated with poor survival (Fig. 1a). Since ADAM17/TACE mediates the cleavage of TβRI to generate TβRI-ICD,^19,20^ we investigated whether *ADAM17/TACE* expression is correlated with *TGFBR1* expression and found a positive correlation between *TGFBR1* and *ADAM17 expression* in both cohorts (Fig. 1b). To map the ADAM17/TACE cleavage site, we established an in vitro assay in which recombinant protein spanning the extracellular domain (ECD) of TβRI was incubated with recombinant TACE. The resulting ECD fragment was analyzed by mass spectrometry, identifying A127/A128 as the C-terminal residue of the cleaved protein. To validate this site, we generated an A128G/I mutant of the recombinant ECD, which displayed markedly reduced cleavage by TACE (Supplementary Fig. 1a, b). Next, we generated a monoclonal antibody targeting the TACE cleavage site to inhibit TβRI-ICD formation. A human single-chain variable fragment (scFv)-library was screened by phage display on recombinant TβRI-ECD133, with alternative panning on a proximal transmembrane peptide to enrich for cleavage site-specific clones. Selected scFvs binding to ECD133 but not to ECD125 were converted into chimeric antibodies with a human variable and a murine constant Fc domain (Supplementary Fig. 2a). To further screen for antibody candidates capable of inhibiting TβRI-ICD formation, we used the reconstituted TβRI-HA-reconstituted PC-3U cell line,^18^ along with an in situ proximity ligation assay (PLA),^28^ which allows visualization of in situ protein‒protein complexes within 40 nm. From this experiment, we selected the candidate mAb 19 for further characterization. To investigate whether mAb19 interferes with TβRI-ICD formation and its binding to p300 in human mCRPC cells, in situ PLA was performed, and the results revealed that mAb19 efficiently decreased the formation of a complex between TβRI-ICD and p300 (Supplementary Fig. 2b). As a model for the in vitro experiments, we selected the PC-3U cell line, which has higher expression of endogenous TβRI than PC-3 cells do.^29^ We observed significant inhibition of the TGFβ-induced invasion of PC-3U cells upon treatment with mAb19 (Supplementary Fig. 2c). Furthermore, we employed biolayer interferometry (BLI) technology to determine the binding affinity of mAb19 for its recombinant target (Supplementary Fig. 2d, e). Furthermore, the Kd values for the binding of mAb19 to cells were determined by fluorescence-activated cell sorting (FACS) in the human prostate epithelial cell lines RWPE-1 and PC-3U and were found to be 7.81 nM and 10.7 nM, respectively (Supplementary Fig. 2f). Importantly, drug target engagement of mAb19 was demonstrated under physiological conditions in clinical materials derived from patients with prostate cancer by a cellular thermal shift assay (CETSA) (Supplementary Fig. 2g). Since treatment strategies that inhibit the kinase activity of TβRI have been reported to cause severe side effects on the heart and proximal aorta,^8^ we investigated whether treatment with mAb19 inhibited canonical TβRI-SMAD signaling in TβRI-HA-reconstituted PC-3U cells.^18^ Treatment with mAb19 did not affect the TGFβ-induced phosphorylation of SMAD2, whereas treatment with the TβRI kinase inhibitor galunisertib effectively blocked SMAD2 phosphorylation, as expected (Fig. 1c–e). To clinically validate the relevance of targeting the complex between TβRI-ICD and p300, the correlations between *TGFBRI* and *EP300* (encoding the p300 protein) or *CREBBP* (encoding the CBP protein) in the public databases SU2C^30^ and TCGA were analyzed. Positive correlations were detected, between the expression of *TGFBRI* and both *EP300* and *CREBBP*, in both prostate adenocarcinoma and metastatic prostate cancer, providing support for the clinical relevance of targeting the identified protein complex (Fig. 2a, b). After characterization of mAb19, in vitro, we investigated the effect of mAb19 in a preclinical orthotopic mCRPC model, including metastasis to regional lymph nodes (Fig. 3a) using aggressive mCRPC PC-3U cells.^18,31^ We previously reported that the expression of *TGFBR1* and *PKCζ* contributes to the lymphatic spread of PC-3U cells in this preclinical orthotopic mCRPC model through production of extracellular matrix, activation of ADAM17/TACE and promotion of lymph angiogenesis.^17,28^ Treatment of tumor-bearing nude-Foxn1^Nu^ mice with galunisertib daily per os (75 mg/kg or 150 mg/kg) or mAb19 at 50  mg/kg twice a week given as intraperitoneal injections (i.p.) significantly inhibited the number of metastases to regional lymph nodes (Fig. 3b) compared with that in the control groups, which were treated with vehicle or isotype-specific mAb. Treatment of tumor-bearing nude-Fox1^Nu^ mice with 50 mg/kg mAb19 i.p. twice a week for 30 days significantly reduced the weight and area of tumors (Fig. 3c, d). Compared with treatment with the isotype-specific IgG control and no treatment, treatment with mAb19 at 10 or 50 mg/kg significantly reduced the weight and area of metastases in regional lymph nodes (Fig. 3c, e). No adverse effects or loss of body weight were observed during the treatment period (Fig. 3f). Using the in situ proximity ligation assay (PLA) technique, to measure complexes between TβRI-ICD and p300, we demonstrated that compared with control antibodies at 10 or 50 mg/kg, treatment with mAb19 significantly reduced the number of nuclear TβRI-ICD-p300 complexes (Fig. 3g, h). Taken together, these data showed that targeting TβRI with a monoclonal antibody prevented the proteolytic cleavage of TβRI and hindered the growth and metastasis of mCRPC in vivo; thus, targeting TβRI-ICD formation could be of therapeutic value. Notably, we observed a positive correlation between the expression of *TGFBRI* and *EP300* as described above (Fig. 2a) in clinically derived prostate adenocarcinoma and metastases. Encouraged by these observations, we decided to develop a fully human, affinity-maturated antibody that can be used clinically.

### Generation and validation of an affinity-matured fully human antibody

A fully human clone19 (humAb19) was produced, and its performance was compared with that of chimeric mAb19. By utilizing an in situ PLA for nuclear TβRI-ICD, we verified that humAb19 maintained its functionality (Supplementary Fig. 3a**)**. To improve the affinity of mAb19 for TβRI, we next carried out affinity maturation by using a phage-Fab library in which exposed amino acids were mutated. The clones with the highest affinity were converted into fully human antibodies, and their binding to PC-3U cells was investigated by FACS to select the best-performing antibody. In this test, we identified the affinity-maturated fully human IgG1 antibody clone F11 (mAbF11) as the best candidate with improved affinity for binding to endogenous TβRI in cells (Supplementary Table S1). The performance of fully human antibodies was then compared with that of the chimeric mAb19 antibody, utilizing the in situ PLA for nuclear TβRI-ICD. mAbF11 showed the greatest ability to hinder nuclear TβRI-ICD, as evaluated by in situ PLA (Supplementary Fig. 3b, c). Compared with a control mAb, treatment of PC-3U cells with mAbF11 resulted in significantly reduced proliferation in a dose-dependent manner (Supplementary Fig. 3d). We also demonstrated by FACS that mAbF11 bound to the human breast carcinoma cell line SK-BR-3 as well as to the human rhabdomyosarcoma cell line RMS13 (Supplementary Fig. 3e). Importantly, we also demonstrated that mAbF11 and mAb19 prevented the cleavage of recombinant TβRI-ECD by TACE (Supplementary Fig. 3f, g).

### Effects of mAbF11 on TGFβ signaling pathways, androgen-regulated genes, EMT and prostate cancer cell invasion

**Fig. 4:**
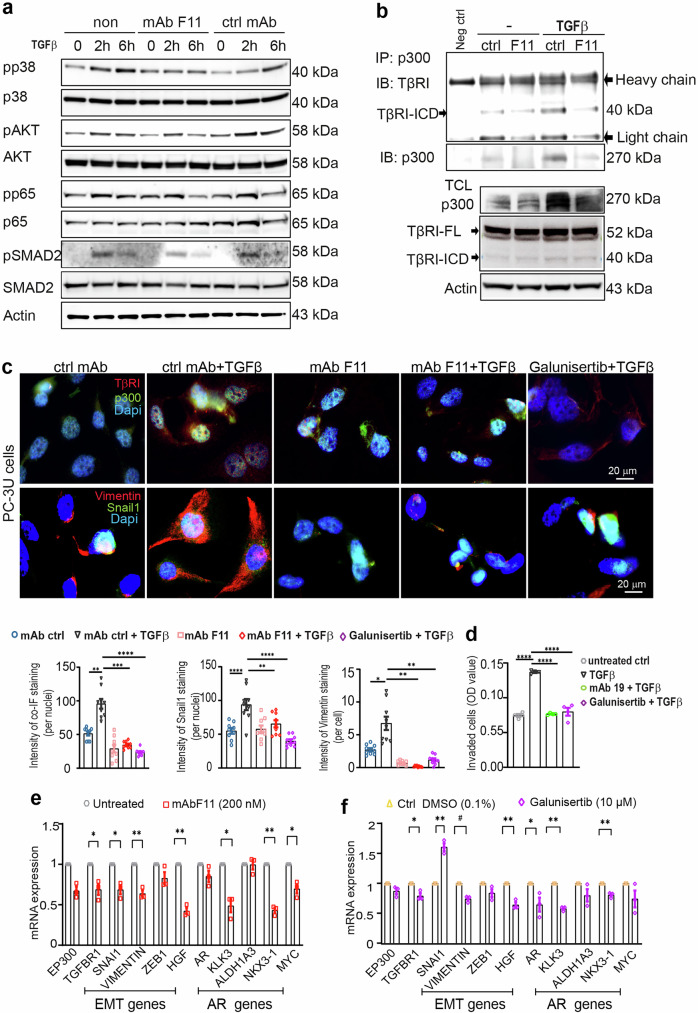
Effects of mAbF11 on TGFβ signaling, EMT, androgen-regulated pathways and invasiveness in mCRPC cells. **a** PC-3U cells were treated with 200 nM mAb F11, isotype control mAb (Ctrl mAb), or PBS (non) and stimulated with TGF-β1 for 2 or 6 h. The cell lysates were subjected to immunoblotting with antibodies against pp38 MAPK, pAKT, pp65, and pSMAD2 and antibodies against the corresponding total proteins. Immunoblotting for β-actin served as loading control. Images from a representative immunoblot experiment. **b** Coimmunoprecipitation (Co-IP) was performed to assess the interaction between TβRI-ICD and p300 in PC-3U cells pretreated with ctrl mAb or mAb F11 for 1 h and then left unstimulated or stimulated with TGF-β1 for 6 h. The arrow indicates the full-length TβRI-ICD or TβRI (TβRI-FL). **c** Upper row: Co-immunofluorescence (Co-IF) staining of TβRI (red) and p300 (green) in PC-3U cells treated as indicated for 48 h. Lower row: Co-IF staining of vimentin (red) and Snail1 (green) in PC-3U cells treated as indicated for 48 h. Nuclei were stained with DAPI (blue). Scale bar, 20 μm. The mean fluorescence intensity of the colocalized TβRI/p300 (left), nuclear vimentin (middle), and cellular Snail (right) signals was quantified on a per-cell basis, presented as mean values ± SEMs. Statistical analysis was performed using one-way ANOVA (with Tukey’s multiple comparisons test) and Welch’s ANOVA for unequal variances. Experiments performed as triplicates in 3 different experiments (*N* *=* 3). **d** Invasion assay: Invaded cells were stained, and the optical density (OD) was quantified. Cells were either left untreated (i.e., without TGFβ1 stimulation), stimulated with TGFβ1, or additionally treated with 200 nM mAb19 or 10 µM galunisertib. Mean values ± SEMs (*N* = 3). Statistical significance was determined by one-way ANOVA. **e** qRT‒PCR analyses of mRNA expression in PC-3U cells treated with mAbF11 **f**, or 10 μM galunisertib; *EP300, TGFBR1*, and EMT-related genes; and *SNAI1, VIM, ZEB1, HGF*, and AR-related genes: *AR, KLK3, ALDH1A3, NKX3-1*, and *MYC*. (*N* = 3). Statistical significance was determined by Student’s *t* test. *P < 0.05, **P < 0.01, ***P < 0.001, ****P < 0.0001. #P close to <0.05

**Fig. 5:**
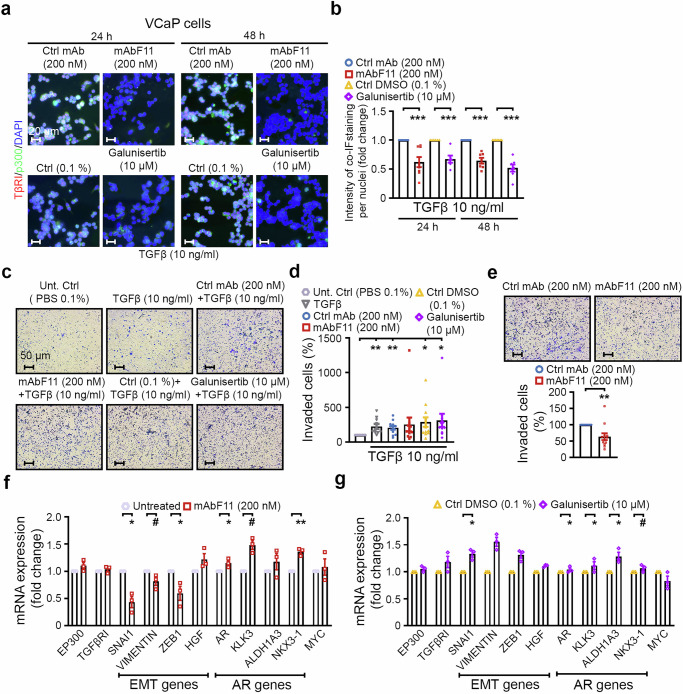
Effects of mAbF11 on TGFβ signaling, EMT and androgen-regulated pathways and invasiveness in androgen-sensitive VCaP cells. **a** Co-immunofluorescence (Co-IF) staining of TβRI (red) and p300 (green) in VCaP cells treated as indicated for 24 h and 48 h. Nuclei are stained with DAPI (blue). Scale bar, 20 μm. **b** The mean fluorescence intensity of the colocalized TβRI/p300 signal within the nuclear region was assessed on a per-cell basis. Image quantification was performed using Fiji software (ImageJ, 1.54 f) and Excel. **c** VCaP cells were treated as indicated: TGFβ1 stimulation only, pretreatment with an isotype control antibody (Ctrl mAb) or mAbF11, or TGFβ1 stimulation in combination with vehicle (0.1% DMSO) or pretreatment with galunisertib. Scale bar, 50 μm. **d** The percentage of invaded cells was quantified in relation to that of the untreated control (Unt. Ctrl). **e** Invasion assay with VCaP cells treated as indicated in the absence of TGFβ1 stimulation. Quantification of invaded cells. qRT‒PCR analyses of mRNA expression in VCaP cells treated with mAbF11 (**f**), or galunisertib (**g**): *EP300 and TGFBR1*; EMT-related genes; *SNAI1, VIM, ZEB1, HGF*, and AR-related genes: *AR, KLK3, ALDH1A3, and NKX3-1;* and *MYC*. In (**b**, **d**–**g**), the mean values ± SEMs were calculated from three independent experiments; in (**b**–**e**) in triplicates (*N* = 3). Statistical significance was determined by Student’s *t* test: *P < 0.05, **P < 0.01, ***P < 0.001, # > close to *P* = 0.05

We investigated the effects of treatment with mAbF11 on the TβRI canonical SMAD2 pathway, as well as on the noncanonical pathways, e.g., activation of the p38 mitogen activated protein kinase (MAPK), phosphatidylinositol 3-kinase (PI3K)/AKT, and nuclear-factor kappa B (NFkB)/p65 pathways, by immunoblotting in PC-3U cells. Treatment with mAbF11 did not appreciably affect these pathways (Fig. 4a). Treatment with mAbF11 resulted in a reduced interaction between TβRI-ICD and p300 in response to TGFβ stimulation of cells, as shown by coimmunoprecipitation, as well as a reduction in the total levels of p300 (Fig. 4b). Next, we investigated the effect of treatment with mAbF11 on the association between endogenous TβRI-ICD and p300 in PC-3U cells by immunofluorescence. Treatment with mAbF11 significantly reduced the levels of TβRI-ICD and p300, as visualized by co-immunofluorescence (Fig. 4c, *upper part*), as well as the expression of the EMT markers vimentin and nuclear Snail (Fig. 4c, *lower part*). Treatment with galunisertib was used for comparison, and we observed that treatment with mAbF11 was as efficient as treatment with galunisertib (Fig. 4c, quantifications shown below the co-immunofluorescence images). In addition, treatment with mAb19 or galunisertib significantly inhibited the TGFβ-induced invasion of PC-3U cells (Fig. 4d). Next, we investigated the effects of treatment with mAbF11 or galunisertib on the mRNA levels of *EP300*, *TGFBR1, and* EMT-related genes, i.e., *SNAI1, VIM*, *ZEB1*, *HGF*, and androgen receptor (AR)-related genes, i.e., *AR, KLK3, ALDH1A3, NKX3-1*, and *MYC*, by quantitative real-time PCR (qRT‒PCR). Treatment with mAbF11 significantly reduced the expression of *TGFBR1; the* EMT-related genes *SNAI1, VIM* and *HGF*; and *KLK3* and *NKX3-1*, as well as *MYC* (Fig. 4e). For comparison, galunisertib treatment significantly decreased the expression of *TGFBR1, VIM, HGF, AR, KLK3* and *NKX3-1* whereas the expression of *SNAI1* was significantly increased (Fig. 4f). To investigate whether treatment with mAbF11 was effective in androgen-sensitive prostate cancer cells, VCaP and C4-2 cells were included in this study. The levels of TβRI were significantly greater in aggressive VCaP, C4-2, PC-3, and PC-3U cells than in less aggressive PNT1A and 22Rv1 cells. Among the investigated cells, PC-3U cells presented the highest level of TβRI, and VCaP cells the highest level of AR (Supplementary Fig. 4a, b). We examined the proliferation of VCaP cells treated with mAbF11 or galunisertib and observed a response to treatment with mAbF11 at 800 nM or galunisertib at 50 μM (Supplementary Fig. 4c). VCaP cells are known to be driven by the *TMPRSS2-ERGa* fusion gene.^32,33^ The effect of treatment with mAbF11 on the levels of endogenous TβRI-ICD and p300 in VCaP cells was found to be significantly reduced, as visualized by co-immunofluorescence. For comparison, the cells were treated with galunisertib, and treatment with mAbF11 was as efficient as treatment with galunisertib (Fig. 5a, b). Stimulation of VCaP cells with TGFβ significantly promoted their invasion, and galunisertib did not inhibit the invasion of these cells, demonstrating that invasion is not dependent on canonical TGFβ signaling via SMADs (Fig. 5c, d). In contrast, treatment with mAbF11 in VCaP cells in the absence of TGFβ stimulation, significantly reduced their invasive ability (Fig. 5e). Analysis of the expression of *EP300*, *TGFBR1*, EMT-related genes and AR-related genes, as well as *MYC*, by qRT‒PCR revealed that treatment of VCaP cells with mAbF11 significantly reduced the expression of *SNAI1* and *ZEB1*, whereas the expression of *AR* and *NKX3-1* significantly increased in response to treatment (Fig. 5f). Treatment with galunisertib resulted in increased expression of *SNAI1, AR, KLK3*, and *ALDH1A3*, indicating that canonical TGFβ signaling via SMADs affects the expression of these genes in androgen-sensitive VCaP cells (Fig. 5g).

Treatment of VCaP cells with mAbF11 or galunisertib reduced the proliferation slightly, albeit not significantly (Supplementary Fig. 4c). We also investigated whether treatment with mAbF11 influenced the nuclear colocalization of TβRI-ICD and p300 induced by TGFβ in another androgen-sensitive cell line, C4-2; no significant effect in response to TGFβ, or by treatment with mAbF11 or galunisertib was observed in these cells (Supplementary Fig. 4d, e). Moreover, neither stimulation with TGFβ, nor treatment with galunisertib affected the invasion of C4-2 cells (Supplementary Fig. 4f). Treatment with mAbF11 significantly reduced the proliferation of C4-2 cells, while treatment with galunisertib had no effect (Supplementary Fig. 4g). Taken together, these data indicate that treatment with mAbF11 decreased the complex between TβRI-ICD and p300 in mCRPC PC-3U cells and androgen-sensitive VCaP cells to significantly reduce invasion. In C4-2 cells, no obvious effects of TGFβ stimulation or treatment with mAbF11 or galunisertib in the presence of TGFβ stimulation were observed on invasion, which is consistent with a previous report that TGFβ does not affect the migration of these cells.^34^ Notably, in the absence of TGFβ stimulation, treatment with mAbF11 significantly reduced proliferation.

On the basis of these data, we decided to further investigate the efficacy of mAbF11 in vivo by employing an orthotopic mCRPC model.

### Effects of mAbF11 in comparison with mAb19 on tumor growth and metastasis in an orthotopic metastatic castration-resistant prostate cancer model

**Fig. 6:**
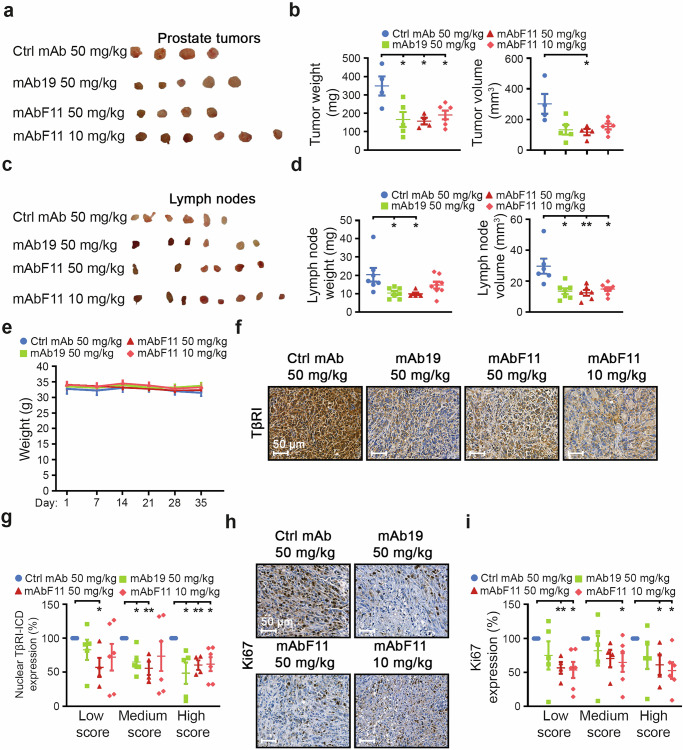
Targeting TGFβRI with mAb19 or mAbF11 leads to decreased tumor growth and metastasis in castration-resistant prostate cancer. **a** Gross morphology of tumors, (**b**) weight and volume of xenograft tumors, (**c**) gross morphology of regional lymph nodes, (**d**) weight and volume of regional lymph nodes, derived from mice treated as indicated with 50 mg/kg Ctrl mAb, 50 mg/kg mAb19, 50 mg/kg mAbF11 or 10 mg/kg mAbF11. **e** Body weight of the mice during treatment. **f** Analyses of expression, and (**g**) quantification of nuclear TβRI-ICDs were performed by using the software QuPath version 0.4.3 for quantification; low (0.2 to 0.4 threshold), medium (0.4–0.6 threshold) and high (0.6 threshold) staining scores were defined by intensity thresholds after cell detection. **h** Analyses of expression and **i** quantification of Ki67 expression in xenograft tumors treated as indicated, as determined by immunohistochemistry and quantification with QuPath. Scale bar, 50 μm. Data presented as means ± SEMs. Statistical significance was determined by Student’s *t* test: *P < 0.05; **P < 0.01

We next investigated the ability of mAbF11 to inhibit growth and metastasis using a preclinical orthotopic mCRPC mouse model in vivo in comparison with mAb19. Compared with treatment with the isotype-specific IgG control (ctrl), treatment with mAb19 or mAbF11 at 10 mg/kg i.p. or at 50 mg/kg i.p. twice a week for 28 days significantly reduced tumor weight. Treatment with mAbF11 at 50 mg/kg significantly reduced tumor weight and volume (Fig. 6a, b). Moreover, compared with treatment with the ctrl mAb, treatment with mAb19 or mAbF11 at 50 mg/kg significantly reduced the weight of metastases in regional lymph nodes, while a reduction in lymph node volume was observed in all treatment groups (Fig. 6c, d). No noticeable side effects or effects on body weight were observed during treatment (Fig. 6e). We previously reported that TβRI expression is promoted by TβRI-ICD^22^ and that TβRI promotes proliferation of mCRPC PC-3U cells.^17^ We found that treatment with mAbF11 significantly reduced the expression of TβRI and proliferation, as determined by the expression of Ki67 determined by immunohistochemistry (IHC), in orthotopic human mCRPC tumors. Interestingly, we also observed a significant reduction in both nuclear TβRI-ICD and cell proliferation, when quantified, with mAbF11 being more efficient than treatment with mAb19 with respect to proliferation (Fig. 6f–i). These data are also consistent with more efficient uptake of mAbF11 than mAb19 in orthotopic tumor tissue when investigated by immunofluorescence (Supplementary Fig. 5a). Moreover, the expression of vimentin, a commonly used marker for TGFβ-induced epithelial‒mesenchymal transition (EMT), was also significantly reduced by treatment with mAbF11 at 10 mg/kg or 50 mg/kg i.p. twice a week (Supplementary Fig. 5b, c). Examination of the morphology of cardiomyocytes revealed no side effects after treatment with mAbF11 at 50 mg/kg i.p. for 4 weeks (Supplementary Fig. 5d). We conclude that mAbF11 inhibits the growth and metastasis of mCRPC in vivo, without noticeable side effects on mouse body weight or cardiomyocyte morphology.

### mAbF11dose-dependently decrease tumor growth and metastasis without affecting heart function

**Fig. 7:**
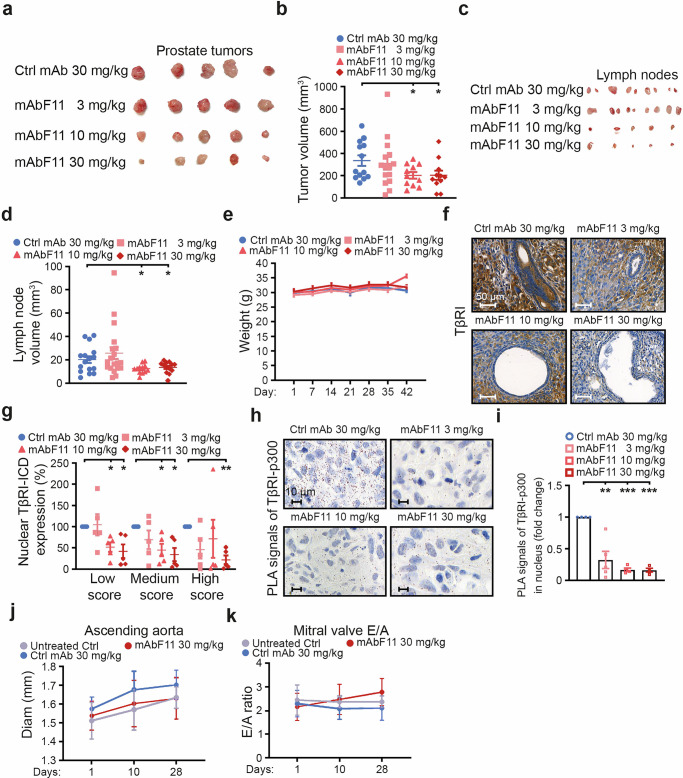
Dose‒response analysis of the effects of mAbF11 on tumor growth and metastasis in castration-resistant prostate cancer and on heart function. **a** Gross morphology and **b** volume of xenograft tumors treated as indicated. **c** Gross morphology and **d** volume of regional lymph nodes, treated as indicated. **e** Body weight during the treatment period. **f** Analyses of expression of TβRI by immunohistochemistry, and (**g**) quantification with QuPath of nuclear TβRI-ICD in xenograft tumors growing in the anterior lobe of the prostate. **h** In situ proximity ligation assay (PLA) of TβRI and p300 to assess the inhibitory effect of mAbF11 on the nuclear TβRI-ICD - p300 complex in vivo, and **i** quantification, in xenograft tumors after 30 mg/kg Ctrl mAb, 3 mg/kg mAbF11, 10 mg/kg mAbF11 or 30 mg/kg mAbF11 treatment as indicated; scale bar, 10 μm. **j** Ultrasound measurement of the diameter of the ascending aorta and **k** on mitral valve ejection, in mice treated for 4 weeks with intraperitoneal injections of 30 mg/kg three times/week or left untreated as indicated. The data are presented as the mean values ± SEMs. Scale bar, 50 μm. Statistical significance was determined by Student’s *t* test: *P < 0.05; **P < 0.01, **P < 0.01

Next, we examined the efficacy of mAbF11 on tumor growth and metastasis in a dose‒response study using an mCRPC model. As the affinity of mAbF11 was improved by affinity maturation, we could lower the highest dose of mAbF11 to 30 mg/kg i.p. twice a week instead of 50 mg/kg i.p. twice a week. Administration of mAbF11 in the orthotopic mCRPC model resulted in dose-dependent and significant decreases in tumor growth and metastases to regional lymph nodes at the two highest doses (10 mg/kg or 30 mg/kg) (Fig. 7a–d), without noticeable side effects or loss of body weight (Fig. 7e). The effects of treatment with mAbF11 at 10 mg/kg or 30 mg/kg led to a significant reduction in nuclear TβRI-ICD as determined by IHC (Fig. 7f, g). Tumor cell proliferation (as determined by Ki67 IHC) was significantly reduced by all doses of mAbF11 (Supplementary Fig. 6a, b). In addition, all doses resulted in a significant decrease in vimentin expression (Supplementary Fig. 6c, d). Moreover, in all treatment groups, a significant decrease in the complex between nuclear TβRI-ICD and p300 was observed after the administration of mAbF11, as visualized by in situ PLA (Fig. 7h, i). To evaluate the possible side effects of treatment with mAbF11 on cardiac function, mice were treated with mAbF11 (30 mg/kg i.p.) three times a week for four weeks, whereafter we measured the proximal aortic diameter with high-frequency ultrasound, as well as the filling of the left ventricle by Doppler. No difference was detected between mice treated with mAbF11 or the isotype-specific control (Fig. 7j, k). On the basis of these data, we conclude that treatment with mAbF11 can prevent tumor growth, EMT and metastasis in an mCRPC model in vivo by reducing nuclear TβRI-ICD complex formation with p300 in a dose-dependent manner. Moreover, no side effects on heart function or the diameter of the proximal aorta were observed.

### Comparison of the efficacy of mAbF11 with that of docetaxel

**Fig. 8:**
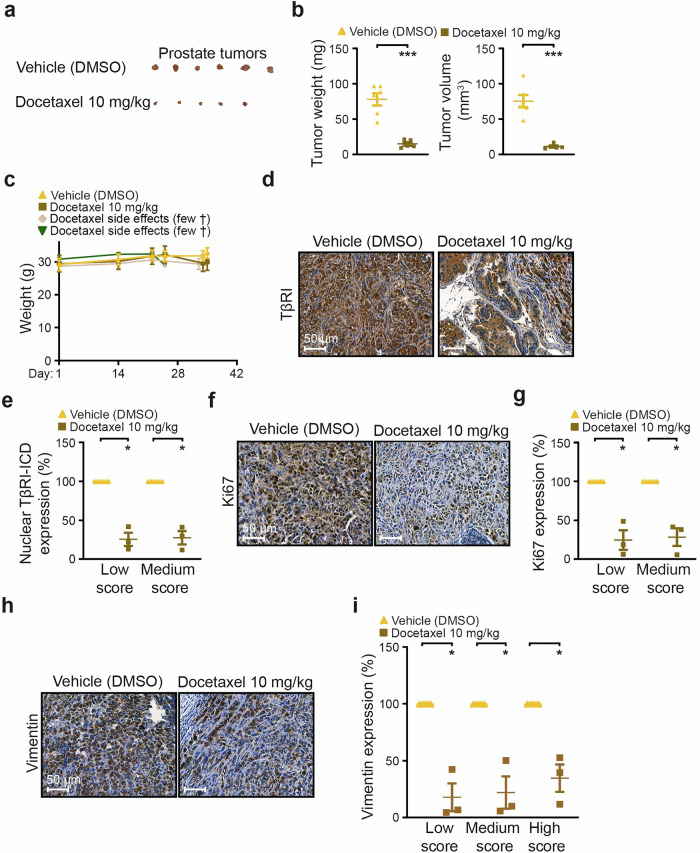
Effect of docetaxel on tumor growth and metastasis in castration-resistant prostate cancer. **a** Gross morphology (**b**) and tumor weight and volume, of xenograft tumors treated as indicated. **c** Body weight of the mice during the treatment period. The cross indicates study termination due to significant body weight loss in some of the mice in the treatment group. **d** Analyses of expression and (**e**) quantification of nuclear TβRI-ICDs. **f** Analysis of expression and (**g**) quantification of Ki67 and of vimentin, (**h**, **i**) as determined by immunohistochemistry and quantification with QuPath software in xenograft tumors from the anterior lobe of the prostate after treatment with vehicle (DMSO) or docetaxel (10 mg/kg, intraperitoneally, twice weekly). Scale bar, 50 μm. Data presented as means ± SEMs. Statistical significance was determined by Student’s *t* test: *P < 0.05; ***P < 0.001

Docetaxel is considered the standard of care for patients with mCRPC.^35^ To simulate clinical practice, we therefore compared the efficacy of treatment with mAbF11 and docetaxel in a preclinical orthotopic mCRPC model. The dose was selected on the basis of previous reports in the literature **(**Supplementary Table 2**)**. Treatment with 10 mg/kg docetaxel significantly decreased tumor volume and weight (Fig. 8a, b). The in vivo study had to be terminated because of severe loss of body weight in 8 out of the 16 mice in the group treated with docetaxel, approximately 2.5 weeks after treatment started, following ethical guidelines (Fig. 8c). Treatment with docetaxel significantly reduced the expression of TβRI, proliferation (assessed by Ki67 expression) and EMT, as assessed by vimentin expression (Fig. 8d–i). Moreover, treatment with mAb19 or mAbF11 inhibited the TGFβ-induced invasion of PC-3U cells in vitro as efficiently as docetaxel did (Supplementary Fig. 7a, b**;** Supplementary Table 3). On the basis of these results, we concluded that treatment with mAbF11 inhibited tumor growth, invasion and metastasis both in vitro and in vivo to a similar extent as treatment with docetaxel but without noticeable side effects.

### Effects of treatment with mAbF11 on TGFβ-induced invasion in triple-negative breast cancer cells and lung metastases in an orthotopic model

**Fig. 9:**
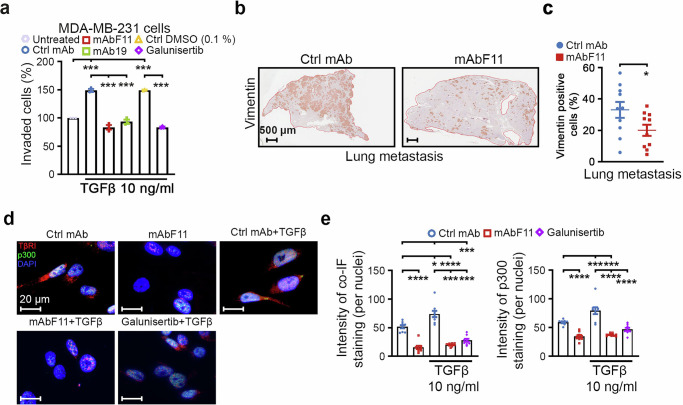
Effects of galunisertib and mAb19/mAbF11 in a murine orthotopic metastatic triple-negative breast cancer model. **a** Invasion assay in human triple-negative breast cancer MDA-MB-231 cells following pretreatment with an isotype-specific monoclonal antibody (Ctrl mAb), mAb19, mAbF11, or galunisertib (10 μM). Controls included non-stimulated cells (untreated) as well as TGFβ1-stimulated cells pretreated with Ctrl mAb. The data are presented as the means of three independent biological replicates ± SEMs (*N* = 3). Statistical significance was determined using Student’s *t* test (*P < 0.01; **P < 0.001). **b** Representative images of lung tissue sections stained for vimentin (brown areas/cells) to visualize lung metastases from mice bearing orthotopic human triple-negative breast cancer tumors. MDA-MB-231 cells were injected into the mammary fat pads, and the mice were treated with either an isotype control antibody (Ctrl mAb; *n* = 10; left) or 30 mg/kg ip mAbF11 twice a week during the study period (*n* = 10; right). Scale bar, 500 μm. **c** Vimentin-positive cells in lung tissue sections were quantified using QuPath software. Statistical significance was determined using the Mann–Whitney U test and Fisher’s exact test (*P ≤ 0.009). **d** Representative images of co-immunofluorescence (co-IF) staining of TβRI (red) and p300 (green) in MDA-MB-231 cells treated as indicated for 48 h. The nuclei were stained with DAPI. Scale bar, 20 μm. **e** The mean fluorescence intensity of the colocalized TβRI/p300 signal or p300 alone was assessed on a per-cell basis using GraphPad Prism software (version 9.4.1) and quantified. The mean values ± SEMs were calculated from three independent experiments in triplicates (*N* = 3). Given that the data did not follow a normal distribution, the nonparametric Kruskal–Wallis’s test was applied for comparisons among multiple groups, ***P < 0.01, ****P < 0.001. For statistical analyses of p300, one-way ANOVA with Tukey’s multiple-comparisons test was used. *n* = 9. Data are presented mean ± SEMs. For co-IF, data are presented as mean ± SEMs. Welch’s one-way ANOVA with Dunnett’s T3 multiple-comparisons test was used for statistical analyses. *n* = 9

To investigate the generality of our findings, we extended our study to include human triple-negative MDA-MB-231 breast cancer cells, as high levels of the TGF-β type I receptor correlate with poor prognosis for patients with invasive breast cancer.^19,36^ We found that treatment with mAbF11 was as efficient as treatment with galunisertib in inhibiting TGFβ-induced invasion in vitro (Fig. 9a). Next, we investigated whether treatment with mAbF11 could reduce the lung metastasis of MDA-MB-231 cells following orthotopic injection into the mammary fat pads of the mice. Notably, the number of lung metastases was significantly lower in the group treated with mAbF11 at 30 mg/kg twice weekly than in the group treated with the Ctrl mAb (Fig. 9b, c). To investigate whether treatment with mAbF11 could affect the TβRI-p300 axis in these cells, co-immunofluorescence staining and imaging for TβRI and p300 were conducted. Treatment with mAbF11 significantly reduced the intensity of co-staining (Fig. 9d, e). No significant effect of stimulation with TGFβ or treatment with mAbF11 was observed on the intensity of Snail1 or vimentin expression (Supplementary Fig. 8a, b). These data indicate that mAbF11 significantly reduces the TβRI-p300 axis in MDA-MB-231 cells, resulting in reduced invasion in vitro and reduced lung metastasis in an orthotopic human triple-negative breast cancer model in vivo.

## Discussion

Metastatic castration-resistant prostate cancer (mCRPC) is a life-threatening disease with few options for effective treatment.^37^ It is therefore crucial to determine the molecular mechanisms driving the proliferation, invasion and metastasis of prostate cancer cells. TGFβ has been recognized as a key driver of EMT, tumor progression and metastasis in several types of solid cancers, e.g., breast carcinoma, lung carcinoma and prostate cancer.^7,15^ Previously we reported that the intracellular domain of TβRI (TβRI-ICD) is generated by proteolytic cleavage in mCRPC cells; PC-3U cells, and that the soluble protein is translocated to the nucleus, where it binds to p300 and to its own gene promoter.^21,22^ Thereby the expression of TβRI is increased leading to a positive feedback loop that amplifies TGFβ signaling activity, together with the production and secretion of THBS1 by modulating the extracellular matrix, to stimulate cell adhesion, and facilitating communication between tumor cells and their surroundings.^16,18^ Intriguingly, THBS1 works in concert with integrin αV (ITGAV), a cell surface adhesion receptor with well-established roles in cancer cell migration, invasion, and metastasis.^18^ ITGAV not only interacts with THBS1 but can also independently activate latent TGFβ, thereby reinforcing the pro-tumorigenic signaling cascade.^16^ Mechanistically, the assembly of a complex comprising TβRI, THBS1, and ITGAV which is localized at the leading edge of migrating mCRPC cells, is enabling an efficient activation of latent TGFβ to orchestrate cellular movements essential for tumor invasion and metastasis.^18^ Collectively, these findings delineate how the spatial localization and molecular interactions of TβRI and/or TβRI-ICD in mCRPC cells is driving key processes underlying tumor progression, linking transcriptional regulation, extracellular matrix remodeling, and migratory signalling.

Several different attempts to block TGFβ in cancer treatment have been evaluated during the past decade, both in preclinical research and in clinical trials, but to date, none have reached clinical usefulness,^9^ partly because of reported severe side effects, including insufficient heart function and dilation of the proximal aorta.^8^ In this study, we describe a novel therapeutic approach for the treatment of mCRPC by using a therapeutic mAb that binds to a specific epitope in TβRI, thereby preventing its proteolytic cleavage by TACE/ADAM17 and nuclear accumulation of TβRI-ICD. We previously reported that nuclear TβRI-ICD binds to p300 and promotes the expression of TβRI and Snail, driving the invasion of cancer cells.^21–23^. In VCaP cells stimulated with TGFβ, neither treatment with galunisertib nor with mAbF11 reduced invasion, which might indicate that the invasiveness of VCaP cells is driven mainly by other pathways, e.g., the androgen-regulated *TMPRSS2-ERG* fusion gene.^32,33^ In castration-resistant PC-3U cells, which lack the *TMPRSS2-ERG* fusion gene,^32,33^ treatment with mAbF11 or galunisertib effectively inhibited the growth of primary tumors in a preclinical orthotopic mCRPC model, as well as metastases to regional lymph nodes, and notably, in the case of mAbF11 without noticeable side effects, such as body weight loss or changes in the diameter of the proximal aorta or heart function, were detected in immunodeficient mice. When treatment with mAbF11 was compared with treatment with docetaxel in vitro, today’s standard treatment of care for patients with mCRPC, mAbF11 was found to be as effective as docetaxel in preventing the TGFβ-induced invasion of mCRPC cells.

TβRI expression in breast carcinoma has been linked to poor patient prognosis.^36^ Inhibiting TGFβ signaling through the expression of a dominant-negative TβRI in human triple-negative MDA-MB-231 breast carcinoma cells resulted in a significant reduction in lung metastasis, demonstrating the important tumor-promoting role of TGFβ signaling in this preclinical model.^38^ To investigate the potential global effect of mAbF11, we included MDA-MB-231 cells in our study. Treatment with mAbF11 reduced the invasion of these aggressive triple-negative breast cancer cells in vitro and inhibited the formation of lung metastases in the corresponding orthotopic model in vivo, suggesting that mAbF11 could be beneficial for the treatment of this cancer subtype.

The lysine acetyltransferase p300 forms a transcriptional complex with the androgen receptor (AR), promoting the activity of oncogenic transcription factor enhanceosomes, which consist of chromatin and epigenetic regulators.^39^ On the basis of this knowledge, specific targeting of p300 for degradation is a potential novel treatment strategy for androgen-sensitive prostate cancer.^5,40,41^ In the present study, we show that treatment with mAbF11 reduces the levels of both p300 and TβRI-ICD and thereby the complex of TβRI-ICD and p300 in mCRPC cells, resulting in reduced growth, invasion, and metastasis to regional lymph nodes. In androgen-sensitive VCaP cells, driven mainly by the TMPRSS2-ERG fusion gene, treatment with mAbF11 reduced TβRI-ICD-p300 complex activity and tumor cell invasion in the absence of TGFβ, whereas in the presence of TGFβ, neither mAbF11 nor galunisertib reduced VCaP invasion, suggesting that invasion of these cells is primarily mediated by other pathways, consistent with previous reports.^32,33^

TGFβ is known to dampen the immune response against cancer cells.^7,11^ Several preclinical studies have shown beneficial treatment treatment effects with a combination of TβRI kinase inhibitors with checkpoint inhibitors, such as antibodies against PD1 or PD-L1, e.g., in genetically defined colorectal cancer models^8,42^ and breast cancer.^43^ A limitation of the present study is that it was performed in immunodeficient mice; thus, no information about the potential effects of treatment with mAb19/mAbF11 on the immune response could be obtained. Moreover, as T cells are known to contribute to metastases,^44^ future studies are needed to investigate whether treatment of mCRPC, triple-negative breast cancer, or other forms of metastatic cancer can be improved by a combination of treatments with mAbF11 and checkpoint inhibitors or other forms of treatment.

In conclusion, we demonstrate that the affinity-matured, fully human mAbF11 prevents the generation of TβRI-ICDs; inhibits the invasion of androgen-sensitive VCaP cells and mCRPC cells in vitro; and suppresses mCRPC growth and metastasis in vivo while preserving physiological TGFβ-SMAD signaling. Moreover, treatment of human breast carcinoma MDA-MB-231 cells resulted in the inhibition of invasion in vitro and lung metastasis in vivo. Targeting the protumorigenic effects of TGFβ by a specific strategy, as demonstrated in this study, presents a potential approach to prevent the growth and metastasis of cancer cells dependent on this pathway, without the risk of causing the deleterious side effects that have been observed when TGFβ signaling is completely inhibited.

## Materials and methods

### Cell culture

The human castration-resistant prostate carcinoma (androgen-independent) PC-3U cell line^29^ is a clone from the original PC-3 cell line (CRL-1435, ATCC). Compared with PC-3 cells, PC-3U cells express higher levels of endogenous TβRI.^29^ PC-3U cells, TGFβR1-deficient PC-3U (A9) cells, TGFβR1-HA-reconstituted PC-3U cells^18^ and RMS13 cells (CRL-2061, ATCC), androgen-sensitive C4-2 cells (CRL-3314, ATCC), 22Rv1 cells (CRL-2505, ATCC) and PNT1A cells (95012614, UK Health Security Agency) were grown in RPMI-1640 media (R0883, Merck), triple-negative human breast cancer cell line MDA-MB-231 cells (CRM-HTB-26, ATCC) in L15 media (11415064, Thermo Fisher Scientific), SK-BR-3 cell line (HTB-30, ATCC), McCoy’s 5a medium (Thermo Fisher, 12330031), and human prostate RWPE-1 cells (CRL-3607, ATCC) in keratinocyte serum-free medium (Thermo Fisher Scientific, 17005042) supplemented with 2 mM glutamine (Merck). Androgen-sensitive VCaP cells (CVCL 2235, ATCC) were grown in Dulbecco’s modified Eagle’s medium (DMEM). All media were supplemented with 10% fetal bovine serum (FBS) (F7524; Merck), 2 mM L-glutamine (G7513; Merck), and 0.1 mg/ml penicillin and streptomycin (P0781; Merck). All the cell lines were maintained at 37 °C in a 5% CO_2_ atmosphere. The cells were starved for 12–18 h in 1% FBS before stimulation with TGFβ1 (10 ng/ml) (Prospec, Ness-Ziona, Israel). The TβRI kinase inhibitor LY2157299 (HY-15150; MedChem Express), i.e., anti-galunisertib and anti-TβRI-targeting antibodies, were added, or not, half an hour before TGFβ stimulation. Galunisertib was solubilized in 0.1% DMSO.

### Antibodies

mAb19 was expressed as a murine chimeric IgG1 consisting of the human variable region of clone 19 and murine IgG1/kappa. mAb clone #4, mAb clone #16, and the isotype control murine antibody specific for fluorescein were expressed in the same manner as mAb19. Affinity maturation of the anti-TβRI antibody clone 19 was performed by CDR mutagenesis at Yumab, Germany, as described in the Supplementary Materials. The clone F11, which showed the highest affinity in functional assays, was selected for further development to generate a fully human IgG1 antibody. A nonbinding IgG1 antibody was used as a human isotype control antibody.

### Immunoblotting

Cells were washed twice in ice-cold phosphate-buffered saline (PBS) and lysed in ice-cold RIPA buffer (150 mM NaCl, 50 mM Tris (pH 8.0), 0.5% (v/v) sodium deoxycholate, 1% (v/v) NP40, and 10% (v/v) glycerol and protease inhibitors). After centrifugation, the supernatants were collected, and protein concentrations were determined by a BCA protein assay kit (23225; Thermo Fisher Scientific). Equal amounts of protein from each total cell lysate were subjected to Mini-PROTEAN TGX gels or CRITERION TGX gels (Bio-Rad, 4569036 and 5671125) followed by transfer onto nitrocellulose membranes and immunoblotting using primary antibodies including pSMAD2 (3108S) and SMAD2 (3103S), pp38 (9211S), p38 (9217S), pAKT (4060S), AKT (2920S), pp65 (3033S), and p65 (8242S), all from Cell Signaling; β-actin (A5441, Sigma), p300 (AF3789, R&D), and ΤβRΙ (235578, Abcam); androgen receptor (MA5-13426, Invitrogen), the dilutions are described in Supplementary Table 4.

### Co-immunoprecipitation (Co-IP)

Co-IP was performed to examine the interaction between p300 and TβRI-ICD in PC-3U cells. Cell lysis was performed as described for immunoblotting. To facilitate detection of the low-abundance TβRI-ICD-associated complex, high-concentration lysates prepared from three 10-cm dishes of cells were used for each immunoprecipitation sample. Equal amounts of protein lysates were incubated overnight at 4 °C with rotation with anti-p300 antibody (Santa Cruz, sc-48343), and normal mouse IgG (Santa Cruz, sc-2025) was used as a negative control. On the following day, pre-washed Protein G Sepharose 4 Fast Flow beads (Cytiva, 17061801) were added and incubated for 90 min at 4 °C. The beads were then washed four times with ice-cold RIPA buffer to minimize non-specific binding. Immunocomplexes were eluted by boiling in SDS sample buffer, separated by SDS–PAGE, and analyzed by immunoblotting. TβRI in the precipitated complexes was detected using an anti-TβRI antibody (Abcam, ab235578).

### Fluorescence activated cell sorting (FACS)

SK-BR-3 (ATCC) and RMS13 (ATCC) cells, fixed or not in 4% PFA, were stained with 400 nM Ctrl mAb, 200 nM mAbF11, and 400 nM mAbF11. The cells were subsequently incubated with donkey APC-conjugated anti-human IgG antibody (Jackson ImmunoResearch 709-136-149) for 1 h on ice. Binding was analyzed with a BD Accuri C6 flow cytometer (BD).

### In vivo experiments

Male athymic nude mice (Hsd:Athymic Nude-Foxn1^nu^) were purchased from Envigo Netherlands (6–8 weeks of age at arrival) and used for all in vivo experiments, including the orthoptic prostate xenograft model, as previously described.^18,31^ Mice were maintained at the animal facility at Umeå University under standard housing conditions. All animal experiments were approved by the Umeå Ethical Review Board in full agreement with the Swedish Ethical Review Act (Approval ID: A7-2018; A26-2022).

The mice were monitored daily, and body weight was measured twice a week. In accordance with the Swedish Ethical Review Act, the animals were sacrificed when they exhibited impaired conditions or sharply lost weight; otherwise, they were sacrificed 30 days after the injection. The tumors and lymph nodes closest to the tumor were dissected and weighed; in untreated mice, 2 lymph nodes were usually detected, whereas in treated mice, the largest lymph node(s) were identified. Small portions of the tumor tissues were frozen in liquid nitrogen for protein and RNA extraction. The remaining tumor tissues and entire lymph nodes were fixed in formalin and embedded in paraffin (FPPE). Morphological evaluation under light microscopy was performed and analyzed by an investigator who was blinded to the treatment status.

For the first experiment, a lower midline incision was made, and 300 000 PC-3U cells in 10 μl of sterile PBS were injected into the right anterior prostate lobe of 19 mice. One week after the injection, the mice were randomized into different treatment groups: Ctrl for galunisertib (*n* = 13), galunisertib 75 mg/kg (*n* = 9), galunisertib 150 mg/kg (12). For treatment with mAbs; Ctrl mAb (50 mg/kg, *n* = 4), mAb19 (50 mg/kg, *n* = 5), mAbF11 (50 mg/kg, *n* = 4), and mAbF11 (10 mg/kg, *n* = 6). In the second in vivo experiment, 300 000 PC-3U cells in 10 μl of sterile PBS were injected into the right anterior prostate lobe of 72 mice. One week after injection, the mice were randomized into six groups that were treated with intraperitoneal injections of the following compounds twice a week for 4 weeks: Ctrl mAb (30 mg/kg, *n* = 13), 3 mg/kg mAbF11 (*n* = 15), 10 mg/kg mAbF11 (*n* = 11), 30 mg/kg mAbF11 (*n* = 11), Ctrl (vehicle DMSO; *n* = 6), or 10 mg/kg docetaxel (HY-B0011, MedChem Express; *n* = 16).

For in vivo studies, suspensions of galunisertib (TargetMol, T2510) were prepared in the following buffers: 1% (w/v) Carboxymethylcellulose sodium (c9481–500 g, Sigma‒Aldrich), 0.25% (v/v) polysorbate 80 (59924–100G-F, Sigma‒Aldrich), and 0.05% (v/v) Dow Corning antifoam (XIAMETER, AFE-1510, E900-Dimethylpolysiloxane).

### Analyses of heart and aorta function

For the histological evaluation of cardiomyocytes, 4 μm FFPE sections were stained with standard hematoxylin and eosin.

Measurements of proximal aortic diameter and left ventricle filling were performed in seven-week-old NudeFoxn1nu mice. Mice treated with control mAb (*n* = 8), mAbF11 (*n* = 8) and PBS as control (*n* = 8). The mice were given 30 mg/kg ctrl mAb or 30 mg/kg mAbF11 as intraperitoneal injections three times a week for four weeks. High-frequency ultrasound was used to assess the proximal aortic diameter and left ventricle filling before treatment started and after ten and 28 days. In short, transthoracic high-frequency ultrasound with an MX550D transducer (Vevo3100, Fujifilm VisualSonics, Toronto, ON, Canada) was used for cardiac imaging. The diameter of the ascending aorta was measured at end-diastole using M-mode, with the aortic lumen and aortic valve clearly visible. Mitral valve inflow velocities were recorded in the four-chamber apical view using Doppler measurements. The *E*/*A* ratio was calculated from early filling velocity (*E*) and atrial filling velocity (*A*) to evaluate diastolic function. During ultrasound measurements, the mice were slightly sedated with 1–2% isoflurane (Attane vet, VM Pharma Stockholm, Sweden) in 800 μl of O_2_. The mice were kept on a temperature-controlled table, with ECG registration and anesthesia adjusted to avoid depression of respiration. Analyses used Vevo LAB 5.6.1 (Fujifilm VisualSonics, Toronto, ON, Canada) and were performed off-line in a blinded manner.

### Orthotopic MDA-MB-231 tumors in the mammary fat pad and lung metastases

Ten-week-old female NXG mice (Janvier Labs, France) were orthotopically inoculated with 1 million human triple-negative MDA-MB-231 breast carcinoma cells in the right fat mammary gland in an injection volume of 100 μL. All the mice were housed and maintained in microisolator cages under specific pathogen-free conditions, and in accordance with Swedish legislation, the in vivo mouse model was adapted from Dominguez et al. ^45^. The mice were randomized to one of the two treatment groups and treated with either an isotype-specific Ctrl mAb (30 mg/kg, *n* = 10) or a 30 mg/kg mAbF11 (*n* = 10) mAbF11 beginning on day 21 after the tumor cells were inoculated until termination of the study. Metastases in the left lung were visualized by vimentin staining on 4 μM FFPE sections. Vimentin is frequently used as a marker to demonstrate when cells undergo EMT during metastasis progression and is therefore often used for staining of metastasis.^46,47^

### Morphology and immunohistochemistry

Immunohistochemistry (IHC) was performed on 4 µm FFPE tissue sections. After rehydration, the sections were treated in a pressure cooker at 95 °C for 15 min in antigen retrieval reagent (Diva Decloaker BC-DV2004MX, Biocare Medical). Endogenous peroxidase activity was blocked using 3% (v/v) H₂O₂ in methanol. After being blocked with goat serum for 30 min at room temperature, the sections were incubated overnight at 4 °C with the following primary antibodies: TGFβRI (1:500; PA5-98192; Thermo Fisher Scientific), Ki67 (1:250; ab92742; Abcam), or vimentin (1:100; MA5-16409; Thermo Fisher Scientific). This was followed by incubation with an HRP-conjugated secondary antibody (K4003; Agilent Technologies) and visualization using DAKO REAL (K500711-2; Agilent Technologies). The sections were then counterstained with hematoxylin.

Digital images were acquired using Pannoramic 250 Flash II (3DHistech, Hungary). The number of positive cells in relation to that of negative cells was quantified using QuPath version 0.4.3 software.^46,47^ The whole tumors on tumor slides were marked in Qupath, and positive cells were subsequently quantified and subdivided into different thresholds: low at 0.2 ( + 1; meaning 0.2–0.4), medium at 0.4 ( + 2; meaning 0.4–0.6) and high at 0.6 ( + 3; meaning > 0.6) expression levels of the target.

Quantification of lung metastases in the orthotopic MDA-MB-231 model was performed using FFPE tissue sections derived from the left lung of the mice, with a primary antibody for the detection of vimentin (anti-vimentin (SP20) ab16700; Abcam; diluted 1:200 in PBS with 5% goat serum). This was followed by the secondary antibody (Bright Vision HRP (Immunologic, nr DPVR110HRP)) and visualization using DAB (Sigma Aldrich, nr D5637). The sections were counterstained with hematoxylin.

The software QuPath version 0.4.3 was used for quantification, and low (0.2–0.4), medium (0.3–0.4) and high (0.4) staining scores were defined by intensity thresholds after cell detection. Here, vimentin (DAB-brown) staining was used for cell detection. The mean DAB optical density (OD) was used to calculate the intensity of nuclear vimentin staining in lung metastases in the MDA-MB-231 orthotopic model. Twenty slides were analyzed, and the whole image was used for evaluation of nuclear intensity.

### Immunofluorescence staining

Cells were seeded on glass coverslips and cultured to 50% confluence. Prior to stimulation, cells were cultured in the media with 1% FBS for starving overnight and then were pretreated with inhibitors or antibodies for 1 h, followed by stimulation with TGFβ1 for the indicated time periods. Detailed treatment conditions are described in the corresponding figure legends. Cells were then fixed with 4% paraformaldehyde, permeabilized with Triton X-100, and blocked with 5% donkey serum. Coverslips were incubated with primary antibodies overnight at 4 °C, followed by fluorophore-conjugated secondary antibodies for 1 h at room temperature. Nuclei were counterstained with DAPI. After washing, coverslips were mounted with Fluoromount-G and imaged using a fluorescence microscope (Axioplan 2, Zeiss) and a digital camera (4742-95, Hamamatsu). Nuclear fluorescence intensity was quantified using Fiji ImageJ 1.54 f.

### Immunofluorescence of tumor tissue slides

Sections of FFPE tumor tissue derived from mice treated with 50 mg/kg Ctrl mAb, 50 mg/kg mAb19 or 50 mg/kg mAbF11 twice a week were rehydrated in deionized H_2_O, treated with Antigen Retrieval Reagent (BC-DV2004MX, Histolab) at 95 °C for 15 min, blocked with 1% BSA and then incubated with a primary antibody against TGFβR1 (PA5-98192, Thermo Fisher Scientific, 1:200, anti-rabbit) overnight at 4 °C. The secondary antibodies used were Alexa Fluor 488 (A3271; Thermo Fisher Scientific; 1:500; anti-rabbit) or APC (709-136-149; 1:200; Novakemi; anti-human) for 1 h at room temperature, after which the slides were rinsed in PBS and incubated with Hoechst (1:500; H1399; Thermo Fisher Scientific) for 5 min at RT before they were mounted with Fluoromount-G (0100-01; AH Diagnostics). Pictures were taken by Carl Zeiss Microscopy GmbH (Zeiss).

### In situ PLA

*An* in situ proximity ligation assay (PLA) was performed according to the protocol for the Duolink PLA probes and Duolink Detection Kit from Merck, as previously described.^18,28^ Briefly, cells were fixed on glass coverslips, blocked and incubated overnight with primary antibodies specific to HA (1:200; 3724S; Cell Signaling) and p300 (1:200; AF3789; R&D Systems). After being washed, the cells were incubated with two sets of probe-conjugated secondary antibodies (DUO92005, DUO92003). Probes were then ligated with a bridging probe in a proximity-dependent manner, which allowed rolling-circle amplification. Finally, interacting proteins could be visualized as fluorescent dots (fluorescence detection kit DUO92007) or red dots (brightfield detection kit DUO92012) under a microscope (Carl Zeiss Microimaging). For brightfield detection, the sections were pretreated with 3% (v/v) H_₂_O_₂_ in methanol to quench endogenous peroxidase activity, and primary antibodies against TGFβR1 (1:500; Thermo Fisher Scientific) and p300 (1:100; AF3789; R&D) were used. Images were taken with a Pannoramic 250 Flash, and the PLA signal was analyzed by using Duolink Image Tool software.

### Invasion assay

Invasion assays were performed using Corning Matrigel-coated transwell chambers according to the manufacturer’s instructions (Fisher Scientific, 11573570). Cells (PC-3U, MDA-MB-231, VCaP, and C4-2) were serum-starved overnight in medium supplemented with 1% FBS and then were treated with indicated antibodies or inhibitors (including mAbF11, mAb19, docetaxel or galunisertib, or vehicle as control) at specified concentrations. Cells were seeded in the upper chamber, and medium containing 5% FBS (PC-3U), 50% FBS (VCaP), 15% FBS (C4-2) was added to the lower chamber as a chemoattractant. After 24 h, invaded cells were fixed with 4% paraformaldehyde, stained with crystal violet, and quantified by counting cells in representative fields.

### Cellular thermal shift assay

A cellular thermal shift assay (CETSA) was conducted^48^ to generate melt and isothermal dose–response curves, allowing determination of the EC50 for mAb19 binding to TGFBRI in tissue extracts. Snap-frozen xenograft tissues (*n* = 5, PC-3U tumors) and human prostate cancer biopsy samples derived from the UCAN collection of clinical material^49^ (*n* = 6) were used for CETSA analysis of TGFBRI. Tissue extracts were prepared by mechanical disruption and freeze–thaw cycles in HEPES buffer, followed by centrifugation to obtain soluble protein fractions. For thermal shift experiments, tissue extracts were incubated with mAb19 (0.2 μM) or PBS control, aliquoted, and subjected to a temperature gradient (37–60 °C). For the isothermal dose–response experiments, the extracts were incubated with serial dilutions of mAb19 and heated to 49 °C. After centrifugation, the soluble fractions were analyzed by immunoblotting using an anti-TGFBR1 antibody (ab31013 Abcam) and chemiluminescence detection. The band intensities were quantified by densitometry and normalized to those of the control samples. The data were analyzed in GraphPad Prism v.10.61, and the values are presented as the mean ± SEM.

### RNA extraction

Cells were grown in Petri dishes until they reached 80% confluence and then starved for 16 h, after which they were treated with mAbF11 (200 nM), galunisertib (10 μM) and 0.1% DMSO for 24 h. The cells were trypsinized, the cell pellet was collected, and total RNA was extracted using an All Prep DNA/RNA/Protein Mini Kit (Qiagen). The concentration of RNA was determined by a Nanodrop (Denovix DS-11). All the samples were normalized to 20 ng/20 µl per reaction and converted to first-strand cDNA using Superscript IV (Invitrogen 18091050).

Gene expression analysis was performed by quantitative real-time (qRT)-PCR by using Fast SYBR Green master mix (Applied Biosystems). Primers used for qRT‒PCR are listed in Supplementary Table 5.

### Statistical analysis

Two-tailed unpaired Student’s *t* tests were performed for comparisons between two groups with a normal distribution. The nonparametric Mann–Whitney U test or Kruskal–Wallis test was used for comparisons among multiple groups, unless otherwise indicated. *P* ≤ 0.05 was considered to indicate statistical significance. Echocardiographic data were analyzed by one-way ANOVA with Tukey’s post hoc test for each separate time point. The error bars represent the standard deviation (SD) of the mean, unless otherwise indicated. Statistical tests and the number of repeats are described in the figure legends.

### Ethics

The present study was approved by the institutional review board and ethical committee of Northern Sweden (Regionala Etikprövningsnämnden, Umeå University, Umeå, Sweden); approval numbers A7-2018; A26-2022. Written informed consent was obtained from the patients after oral information was given by the staff.

## Supplementary information

Supplemental material

## Data Availability

All the raw data supporting conclusions are included in the manuscript and in Supplementary Material.

## References

[1] Ferlay, J. et al. Cancer statistics for the year 2020: an overview. *Int. J. Cancer* **149**, 778–789 (2021). 10.1002/ijc.33588 33818764

[2] Esfahani, M., Ataei, N. & Panjehpour, M. Biomarkers for evaluation of prostate cancer prognosis. *Asian Pac. J. Cancer Prev.* **16**, 2601–2611 (2015). 25854335 10.7314/apjcp.2015.16.7.2601

[3] Bray, F. et al. Global cancer statistics 2022: GLOBOCAN estimates of incidence and mortality worldwide for 36 cancers in 185 countries. *CA Cancer J. Clin.* **74**, 229–263 (2024). 38572751 10.3322/caac.21834

[4] Lonergan, P. E. & Tindall, D. J. Androgen receptor signaling in prostate cancer development and progression. *J. Carcinog.* **10**, 20 (2011). 21886458 10.4103/1477-3163.83937 PMC3162670

[5] He, Y. et al. Targeting signaling pathways in prostate cancer: mechanisms and clinical trials. *Signal. Transduct. Target Ther.* **7**, 198 (2022). 35750683 10.1038/s41392-022-01042-7 PMC9232569

[6] Tsaur, I. et al. Aggressive variants of prostate cancer - Are we ready to apply specific treatment right now? *Cancer Treat. Rev.* **75**, 20–26 (2019). 30875581 10.1016/j.ctrv.2019.03.001

[7] Massague, J. & Sheppard, D. TGF-beta signaling in health and disease. *Cell* **186**, 4007–4037 (2023). 37714133 10.1016/j.cell.2023.07.036 PMC10772989

[8] Anderton, M. J. et al. Induction of heart valve lesions by small-molecule ALK5 inhibitors. *Toxicol. Pathol.* **39**, 916–924 (2011). 21859884 10.1177/0192623311416259

[9] Teixeira, A. F., Ten Dijke, P. & Zhu, H. J. On-target anti-tgf-beta therapies are not succeeding in clinical cancer treatments: What are remaining challenges? *Front. Cell Dev. Biol.* **8**, 605 (2020). 32733895 10.3389/fcell.2020.00605 PMC7360684

[10] Ivanovic, V., Melman, A., Davis-Joseph, B., Valcic, M. & Geliebter, J. Elevated plasma levels of TGF-beta 1 in patients with invasive prostate cancer. *Nat. Med.* **1**, 282–284 (1995). 7585049 10.1038/nm0495-282

[11] Derynck, R., Turley, S. J. & Akhurst, R. J. TGFbeta biology in cancer progression and immunotherapy. *Nat. Rev. Clin. Oncol.* **18**, 9–34 (2021). 32710082 10.1038/s41571-020-0403-1 PMC9721352

[12] Heldin, C. H. & Moustakas, A. Signaling receptors for TGF-beta family members. *Cold Spring Harb. Perspect. Biol.* **8**, a022053 (2016). 10.1101/cshperspect.a022053 PMC4968163 27481709

[13] Mu, Y., Gudey, S. K. & Landstrom, M. Non-Smad signaling pathways. *Cell Tissue Res.* **347**, 11–20 (2012). 21701805 10.1007/s00441-011-1201-y

[14] Deng, Z. et al. TGF-beta signaling in health, disease, and therapeutics. *Signal. Transduct. Target Ther.* **9**, 61 (2024). 38514615 10.1038/s41392-024-01764-w PMC10958066

[15] Teixeira, A. F., Wang, Y., Iaria, J., Ten Dijke, P. & Zhu, H. J. Simultaneously targeting extracellular vesicle trafficking and TGF-beta receptor kinase activity blocks signaling hyperactivation and metastasis. *Signal. Transduct. Target Ther.* **8**, 456 (2023). 38105247 10.1038/s41392-023-01711-1 PMC10725874

[16] Derynck, R. & Budi, E. H. Specificity, versatility, and control of TGF-beta family signaling. *Sci. Signal.* **12**, eaav5183 (2019). 10.1126/scisignal.aav5183 PMC6800142 30808818

[17] Song, J. et al. The ubiquitin-ligase TRAF6 and TGFbeta type I receptor form a complex with Aurora kinase B contributing to mitotic progression and cytokinesis in cancer cells. *EBioMedicine* **82**, 104155 (2022). 35853811 10.1016/j.ebiom.2022.104155 PMC9386726

[18] Mu, Y. et al. The TbetaRI promotes migration and metastasis through thrombospondin 1 and ITGAV in prostate cancer cells. *Oncogene* **43**, 3321–3334 (2024). 39304722 10.1038/s41388-024-03165-3 PMC11534692

[19] Mallikarjuna, P., Raviprakash, T. S., Aripaka, K., Ljungberg, B. & Landstrom, M. Interactions between TGF-beta type I receptor and hypoxia-inducible factor-alpha mediates a synergistic crosstalk leading to poor prognosis for patients with clear cell renal cell carcinoma. *Cell Cycle* **18**, 2141–2156 (2019). 31339433 10.1080/15384101.2019.1642069 PMC6986558

[20] Liu, C., Xu, P., Lamouille, S., Xu, J. & Derynck, R. TACE-mediated ectodomain shedding of the type I TGF-beta receptor downregulates TGF-beta signaling. *Mol. Cell* **35**, 26–36 (2009). 19595713 10.1016/j.molcel.2009.06.018 PMC2740991

[21] Mu, Y. et al. TRAF6 ubiquitinates TGFbeta type I receptor to promote its cleavage and nuclear translocation in cancer. *Nat. Commun.* **2**, 330 (2011). 21629263 10.1038/ncomms1332 PMC3113296

[22] Gudey, S. K. et al. TRAF6 stimulates the tumor-promoting effects of TGFbeta type I receptor through polyubiquitination and activation of presenilin 1. *Sci. Signal.* **7**, ra2 (2014). 24399296 10.1126/scisignal.2004207

[23] Song, J. et al. APPL proteins promote TGFbeta-induced nuclear transport of the TGFbeta type I receptor intracellular domain. *Oncotarget* **7**, 279–292 (2016). 26583432 10.18632/oncotarget.6346 PMC4807998

[24] Schultz-Cherry, S., Lawler, J. & Murphy-Ullrich, J. E. The type 1 repeats of thrombospondin 1 activate latent transforming growth factor-beta. *J. Biol. Chem.* **269**, 26783–26788 (1994). 7929414

[25] Firlej, V. et al. Thrombospondin-1 triggers cell migration and development of advanced prostate tumors. *Cancer Res.* **71**, 7649–7658 (2011). 22037878 10.1158/0008-5472.CAN-11-0833

[26] Abida, W. et al. Genomic correlates of clinical outcome in advanced prostate cancer. *Proc. Natl. Acad. Sci. USA* **116**, 11428–11436 (2019). 31061129 10.1073/pnas.1902651116 PMC6561293

[27] Fenor de la Maza, M. D. et al. Immune biomarkers in metastatic castration-resistant prostate cancer. *Eur. Urol. Oncol.* **5**, 659–667 (2022). 35491356 10.1016/j.euo.2022.04.004 PMC7617991

[28] Soderberg, O. et al. Characterizing proteins and their interactions in cells and tissues using the in situ proximity ligation assay. *Methods* **45**, 227–232 (2008). 18620061 10.1016/j.ymeth.2008.06.014

[29] Franzen, P., Ichijo, H. & Miyazono, K. Different signals mediate transforming growth factor-beta 1-induced growth inhibition and extracellular matrix production in prostatic carcinoma cells. *Exp. Cell Res.* **207**, 1–7 (1993). 7686495 10.1006/excr.1993.1156

[30] Robinson, D. et al. Integrative Clinical Genomics of Advanced Prostate Cancer. *Cell* **162**, 454 (2015). 28843286 10.1016/j.cell.2015.06.053

[31] Zang, G., Mu, Y., Gao, L., Bergh, A. & Landstrom, M. PKCzeta facilitates lymphatic metastatic spread of prostate cancer cells in a mice xenograft model. *Oncogene* **38**, 4215–4231 (2019). 30705401 10.1038/s41388-019-0722-9 PMC6756056

[32] Tomlins, S. A. et al. Recurrent fusion of TMPRSS2 and ETS transcription factor genes in prostate cancer. *Science* **310**, 644–648 (2005). 16254181 10.1126/science.1117679

[33] Tomlins, S. A. et al. Role of the TMPRSS2-ERG gene fusion in prostate cancer. *Neoplasia* **10**, 177–188 (2008). 18283340 10.1593/neo.07822 PMC2244693

[34] Miles, F. L., Tung, N. S., Aguiar, A. A., Kurtoglu, S. & Sikes, R. A. Increased TGF-beta1-mediated suppression of growth and motility in castrate-resistant prostate cancer cells is consistent with Smad2/3 signaling. *Prostate* **72**, 1339–1350 (2012). 22228025 10.1002/pros.22482

[35] Sweeney, C. J. et al. Chemohormonal therapy in metastatic hormone-sensitive prostate cancer. *N. Engl. J. Med.* **373**, 737–746 (2015). 26244877 10.1056/NEJMoa1503747 PMC4562797

[36] de Kruijf, E. M. et al. The prognostic role of TGF-beta signaling pathway in breast cancer patients. *Ann. Oncol.* **24**, 384–390 (2013). 23022998 10.1093/annonc/mds333

[37] McManus, H. D. et al. Navigating therapeutic sequencing in the metastatic castration-resistant prostate cancer patient journey. *Prostate Cancer Prostatic Dis.* **28**, 672–683 (2024). 10.1038/s41391-024-00906-z PMC12003708 39420184

[38] Padua, D. et al. TGFbeta primes breast tumors for lung metastasis seeding through angiopoietin-like 4. *Cell* **133**, 66–77 (2008). 18394990 10.1016/j.cell.2008.01.046 PMC2390892

[39] Welti, J. et al. Targeting the p300/CBP axis in lethal prostate cancer. *Cancer Discov.* **11**, 1118–1137 (2021). 33431496 10.1158/2159-8290.CD-20-0751 PMC8102310

[40] Zhang, D. et al. Discovery of a peptide proteolysis-targeting chimera (PROTAC) drug of p300 for prostate cancer therapy. *EBioMedicine* **105**, 105212 (2024). 38954976 10.1016/j.ebiom.2024.105212 PMC11261775

[41] Luo, J. et al. Targeting histone H2B acetylated enhanceosomes via p300/CBP degradation in prostate cancer. *Nat. Genet* **57**, 2468–2481 (2025). 41044247 10.1038/s41588-025-02336-6 PMC12513837

[42] Tauriello, D. V. F. et al. TGFbeta drives immune evasion in genetically reconstituted colon cancer metastasis. *Nature* **554**, 538–543 (2018). 29443964 10.1038/nature25492

[43] Mariathasan, S. et al. TGFbeta attenuates tumour response to PD-L1 blockade by contributing to exclusion of T cells. *Nature* **554**, 544–548 (2018). 29443960 10.1038/nature25501 PMC6028240

[44] Neff, E. P. Models matter in metastasis. *Lab Anim.* **46**, 3 (2016). 10.1038/laban.1170 27991866

[45] Dominguez, C., McCampbell, K. K., David, J. M. & Palena, C. Neutralization of IL-8 decreases tumor PMN-MDSCs and reduces mesenchymalization of claudin-low triple-negative breast cancer. *JCI Insight* **2**, e94296 (2017). 10.1172/jci.insight.94296 PMC5752275 29093275

[46] Liu, S. et al. High vimentin expression associated with lymph node metastasis and predicated a poor prognosis in oral squamous cell carcinoma. *Sci. Rep.* **6**, 38834 (2016). 27966589 10.1038/srep38834 PMC5155220

[47] Kokkinos, M. I. et al. Vimentin and epithelial-mesenchymal transition in human breast cancer-observations in vitro and in vivo. *Cells Tissues Organs* **185**, 191–203 (2007). 17587825 10.1159/000101320

[48] Martinez Molina, D. et al. Monitoring drug target engagement in cells and tissues using the cellular thermal shift assay. *Science* **341**, 84–87 (2013). 23828940 10.1126/science.1233606

[49] Glimelius, B. et al. U-CAN: a prospective longitudinal collection of biomaterials and clinical information from adult cancer patients in Sweden. *Acta Oncol.* **57**, 187–194 (2018). 28631533 10.1080/0284186X.2017.1337926

