# Supplementary material for: Targeting oncogenic TβRI signaling inhibits androgen-independent prostate cancer growth and metastasis
Source: Signal Transduct Target Ther. 2026 Jun 17;11:238. doi: 10.1038/s41392-026-02737-x (PMC13272619; doi:10.1038/s41392-026-02737-x)
Supplement: Supplementary file 1 — Supplemental material [file 41392_2026_2737_MOESM1_ESM.docx]

Supplementary Materials for

**Targeting oncogenic TβRI signaling inhibit androgen-independent prostate cancer growth and metastasis**

Per Flodbring Larsson^1^, Alexej Schmidt^1^, Yabing Mu^1^, Guangxiang Zang^1^**,** Jie Song^1^, Vishnupriya Gajavilli^1^, Junting Tao^1^, **Olena Rakhimova^1^, Madelene Ericsson^1^, Karthik Aripaka^1^, Sofia Halin Bergström^1^,** Wei Yuan^2^, Denisa Bogdan^2^, Aaron (Huairen) Zhang^2^, Jon Welti^2^, Anders Bergh^1^, Johann de Bono^2^, Carl-Henrik Heldin^3^, **Maréne Landström^1^***

.

Correspondence to: [Marene.Landstrom@umu.se](mailto:Marene.Landstrom@umu.se)

**This PDF file includes:**

Materials and Methods for Supplementary Figures S1b, S2a, S2d,e, S3d,e, Table 1,2,3,4,5.

Figure and Figure legends for Supplementary Figure S1 to S8

Supplementary Table 1 with legend

Supplementary Table 2 with legend

Supplementary Table 3 with legend

Supplementary Table 4 with legend

Supplementary Table 5 with legend

Materials and Methods

For the Supplementary Figures and Supplementary Table 1 as indicated below.

**Fig. S1 b**

**TACE cleavage assay:**

Recombinant TGFβR1133-myc-His (13 µg) or the corresponding mutants (A128G or A128I) were incubated with 300 ng recombinant TACE/ADAM17 (R&D Systems, Cat. #930-ADB) in TACE cleavage buffer (50 mM Tris, pH 7.4, 2 mM CaCl₂, 0.05% Tween-20) to a final volume of 30 µl and digested overnight at 28 °C. After digestion, NuPAGE LDS sample buffer (4×, Thermo Fisher, Cat. #NP0007) and reducing agent (10×, Thermo Fisher, Cat. #NP0009) were added to one-third of the reaction volume, adjusted with PBS, and proteins were denatured by heating at 96 °C for 7 min. Samples were resolved on a 4–12% Bis-Tris NuPAGE gel (Thermo Fisher, Cat. #NP0335BOX) using MES running buffer (Thermo Fisher, Cat. #NP0002), and protein bands were visualized by staining with PageBlue (Thermo Fisher, Cat. #24620)

**Fig. S2 a**

**Periplasmatic expression of TGFβRI-ECD-133-myc-(His)_6_ and TGFbRI-ECD-125-myc-(His)_6_.**

The genes encoding the extracellular domain (ECD) of human TGFβRI were cloned into the pOPE101 plasmid (GenBank accession no. Y14585), which enables periplasmatic expression in *E. coli*. The sense primer S-huTβRI-NcoI-25-pOPE (5′-gaatagggccatggcggcgctgctcccgggggcg-3′) and antisense primers AS-huTβRI-125BamHI-pOPE (5′-ccgatagggatccttccacaggaccaaggccagg-3′) or AS-huTβRI-133BamHI-pOPE (5′-ccgatagggatcctggtccagcaatgacagctgc-3′) were used to generate constructs encoding the ECD spanning amino acids 25–125 and 25–133, respectively. PCR products were cloned into the vector using *NcoI* and *BamHI* restriction sites.

The resulting pOPE101 constructs, encoding myc- and (His)₆-tagged TGFβRI-ECD, were transformed into *E. coli* XL10-Gold (Stratagene, USA). Transformants were selected on LB agar plates containing 100 µg/ml carbenicillin, 12.5 µg/ml tetracycline, and 0.1 M glucose. For protein expression, overnight cultures in selective LB medium were diluted into fresh selective medium and grown at 37 °C until reaching an optical density at 600 nm (OD₆₀₀) of 0.6. Protein expression was induced with 75 µM IPTG, followed by incubation at 24 °C for 12 h with shaking (230 rpm).

For periplasmatic isolation of the proteins, bacterial pellets were resuspended in 1/16 culture volume of ice-cold spheroplast solution (50 mM Tris-HCl, pH 8.0, 20% sucrose, 1 mM EDTA) and incubated with gentle shaking for 1 h at 4 °C. The suspension was centrifuged at 30,000 × g for 1 h at 4 °C, and the supernatant (periplasmatic extract) was dialyzed twice overnight against 5 L PBS at 4 °C. The extract was filtered through a 0.45 µm membrane, adjusted to 0.5 M NaCl and 20 mM imidazole, and loaded onto a Ni-NTA column (GE Healthcare, USA). Bound proteins were eluted with PBS containing 0.5 M NaCl and 0.5 M imidazole. Protein-containing fractions were dialyzed twice overnight against 2 L PBS at 4 °C, adjusted to 1 mg/ml, aliquoted, and stored at –80 °C.

Protein purity and integrity were assessed by Coomassie-stained SDS-PAGE and immunoblotting using the c-Myc-specific monoclonal antibody 9E10 or a polyclonal goat antibody recognizing the ECD of human TGFβRI (R&D Systems, Cat. #AF3025).

**Fig. S2 d,e**

**Determination of Kd by Bio-Layer Interferometry (BLI)**

Binding kinetics were determined using bio-layer interferometry (BLI) on a BLItz system (FortéBio, USA; now Explore Octet N1, Sartorius). Antigen immobilization was performed on Ni-NTA biosensor tips (FortéBio, Cat. #18-5101). All dilutions, including those of TGFβRI antigen, antibody, and baseline/wash buffers, were prepared in PBS supplemented with 10 µg/ml biocytin (Sigma-Aldrich, Cat. #B4261).

The target antigen was loaded onto biosensors using 4 µl at a concentration of 0.5 mg/ml, followed by antibody loading with 4 µl at the indicated concentrations. Control measurements were carried out either at the second-highest concentration in the absence of antigen or with an isotype-matched murine control antibody. Control responses were subtracted from experimental data.

Assay steps were performed using the following conditions: initial baseline, 30 s (4 µl); antigen loading, 120 s (4 µl); baseline, 80 s (200 µl); association, 120 s (4 µl); and dissociation, 120 s (200 µl).

**Affinity improvement (Yumab GmbH, Braunschweig, Germany)**

A CDR mutation Fab-library based on modelled exposed amino acids of the parental Fab clone 19 was constructed. Enrichment of phages was performed in four panning rounds with decreasing concentration of the biotinylated target antigen ECD_133_-huFc (from 50 nM to 0.05 nM). In order to avoid cross-reactivity to the most homologue Activin-like kinase (ALK5) target protein, all panning rounds were performed in presence of soluble human ALK1, ALK4, ALK7-murine Fc and human Fc. 1536 random Fab clones were sequenced, of which the binding of 253 unique clones were assessed for the lowest Koff in BLI (Bio-layer interferometry), followed by the production of 48 Fab clones selected for the lowest Kd and signal to noise ratio. 25 candidates were converted into fully human IgG1 and ranked according to the lowest IC_50_ and Kd (as determined by binding in ELISA and BLI against ECD_133_-huFc). The monoclonal antibody (mAb) F11 (7.6 x 10^-9^ M) had a three times higher affinity compared to the original mAb 19 (2.2 x 10^-8^ M).

**Affinity maturation (Yumab GmbH, Braunschweig, Germany)**

A CDR-mutated Fab library was generated based on modeled solvent-exposed amino acids of the parental Fab clone 19. Phage enrichment was performed over four rounds of panning using decreasing concentrations of the biotinylated target antigen ECD133-huFc (from 50 nM to 0.05 nM). To minimize cross-reactivity with homologous receptors, all panning rounds were carried out in the presence of soluble human Alk1-Fc, Alk4-Fc, Alk7-Fc, and human Fc.

In total, 1 536 random Fab clones were sequenced, and binding of 253 unique clones was analyzed by bio-layer interferometry (BLI) to determine dissociation rates (koff). From these, 48 Fab clones with the lowest apparent Kd values and highest signal-to-noise ratios were produced. Twenty-five candidates were subsequently converted into fully human IgG1 and ranked according to their IC₅₀ and K_d values, as determined by ELISA and BLI binding assays against ECD133-huFc.

The monoclonal antibody (mAb) F11 exhibited a three-fold higher affinity (Kd = 7.6 × 10⁻⁹ M) compared with the original mAb 19 (Kd = 2.2 × 10⁻⁸ M).

**Supplementary Table 1**

**Determination of Kd and EC₅₀ values by flow cytometry (Truly Translational, Lund, Sweden)**

Human prostate cancer PC-3U cells were cultured in RMPI-1640 medium supplemented with 10% FBS and 1% penicillin/streptomycin at 37 °C with 5% CO₂. Cells were detached using trypsin/EDTA, resuspended in culture medium, and kept on ice during all subsequent sample preparation and washing steps using pre-cooled buffers.

To evaluate antibody potency, serial dilutions of antibody candidates were prepared in PBS containing 1% BSA in 96-well V-bottom plates, yielding final concentrations in the cell suspensions of 0.003, 0.01, 0.03, 0.1, 0.3, 1, 3, 10, 30, and 100 µg/ml.

For each sample, 5 × 10⁴ cells were collected in FCS buffer (PBS with 1% BSA), blocked with human Fc block in the presence of a viability dye for 10 min at 4 °C, and washed. Cells were then fixed and permeabilized with Cytofix/Cytoperm solution (BD Biosciences, Cat. #554714) for 20 min at 4 °C. After washing, cells were incubated with the antibody dilutions for 30 min at 4 °C, followed by staining with a secondary antibody for 30 min at 4 °C. Single-staining controls were included to correct for fluorescence spillover.

Finally, cells were washed with Perm/Wash solution, resuspended in FCS buffer, and analyzed on a CytoFLEX flow cytometer (Beckman Coulter) at a high flow rate (60 µl/min)

**Fig. S3 c**

**Fluorescence-activated cell sorting (FACS)**

The RMS13 cell line (ATCC, CRL-2061) is a fibroblast-like line derived from a 17-year-old male patient with rhabdomyosarcoma. Cells were cultured in RPMI-1640 medium supplemented with 10% FBS (Sigma, Cat. #F7524), 2 mM L-glutamine (Sigma, Cat. #7513), and penicillin/streptomycin (10,000 U/0.1 mg/ml). According to Proteinatlas.org, TGFβRI RNA expression in RMS13 cells, as normalized transcript per million (nTPM), is 132.

The SK-BR-3 cell line (ATCC, HTB-30) is an epithelial adenocarcinoma line derived from a 43-year-old breast carcinoma patient. Cells were cultured in McCoy’s 5A medium (Thermo Fisher, Cat. #12330031) supplemented with 10% FBS (Sigma, Cat. #F7524), 2 mM L-glutamine (Sigma, Cat. #7513), and penicillin/streptomycin (10,000 U/0.1 mg/ml). TGFβRI RNA expression in SK-BR-3 cells is 118 nTPM.

Adherent cells were cultured until 90–100% confluency. After approximately four passages, cells were detached with trypsin (Sigma-Aldrich, T3925) for 7 min at 37 °C, neutralized with 10% FBS-containing medium, and counted. Cells were washed with FACS buffer (PBS with 0.5% FBS). A fraction of cells was fixed in 4% paraformaldehyde for 30 min on ice.

For staining, cells were washed and incubated with the indicated primary antibodies for 1 h on ice. Following another wash, cells were incubated with a donkey anti-human IgG APC-conjugated secondary antibody (Jackson ImmunoResearch, Cat. #709-136-149) for 1 h on ice. Binding was analyzed using an Accuri flow cytometer, recording 10 000 cells per gated population.

**Fig. S3 g**

**TACE cleavage competition assay**

Four µg of recombinant TGFβRI-ECD133-huFc was pre-incubated in TACE cleavage buffer with titrated amounts of mAb for 1 h. Subsequently, 100 ng of recombinant TACE was added, resulting in final mAb concentrations ranging from 5.5 µM to 22 nM. For negative control, Palivizumab was included at 5.5 µM.

Samples were incubated for 25 h at 28 °C and then analyzed by SDS-PAGE, followed by staining with PageBlue. The full-length TGFβRI-ECD133-huFc has a calculated molecular weight of 38 kDa; however, the apparent molecular weight is higher due to N-glycosylation at amino acid 45 within the ECD. Upon TACE cleavage, the Fc domain is released, yielding a fragment of calculated 26 kDa (indicated by an arrow).

**Fig. S3 d, S4 c,g**

**Growth curves were determined by using IncuCyte SX5 (Sartorius, UK).**

PC-3U cells were seeded at 2000 cells/well, VCaP cells were seeded at 8000 cells/well and C4-2 cells were seeded 4000 cells/well of 96 well plate and grown overnight in 37 °C. After overnight, PC-3U cells were treated with 200-800 nM Ctrl mAb or 200-800 nM mAbF11 in RPMI 1640 with 1% FBS for 72 h. As controls, PC-3U cells were grown in RPMI 1640 with either 10 % or 1% FBS. VCaP cells were treated with 800 nM Ctrl mAb, 800 nM mAbF11, 0.1 % Ctrl (DMSO) and 50 µM galunisertib in DMEM with 10 % FBS for 96 h. C4-2 cells were treated with 200 nM Ctrl mAb, 200 nM mAbF11, 0.1 % Ctrl (DMSO) and 50 µM galunisertib in RPMI 1640 with 10 % FBS for 72 h. Cell proliferation was measured using IncuCyte (Sartorius).

Figure S1.


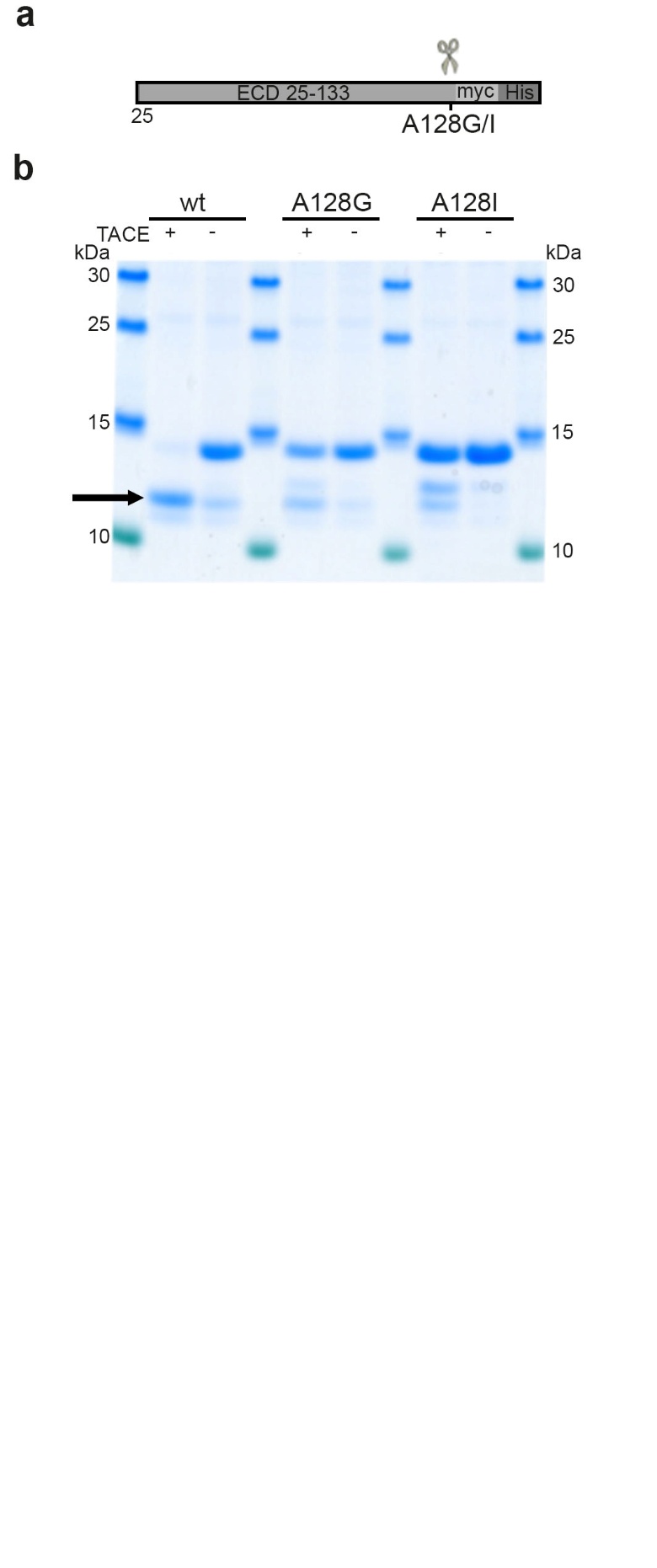


Supplementary Figure S1.

Schematic representation of the recombinant TGFβRI-ECD-133-myc(His)_6_ protein, and identification of an ADAM17/TACE-induced cleavage site.

**a,** Schematic representation of the recombinant TGFβRI-ECD-133-myc(His)_6_ protein with the point mutations depicted at amino acid position 128. **b,** Analysis by SDS-PAGE of wt and A128G and A128I mutated recombinant TGFβRI-ECD-133-myc(His)_6_ proteins, before and after treatment with TACE.

Supplementary Figure S2.


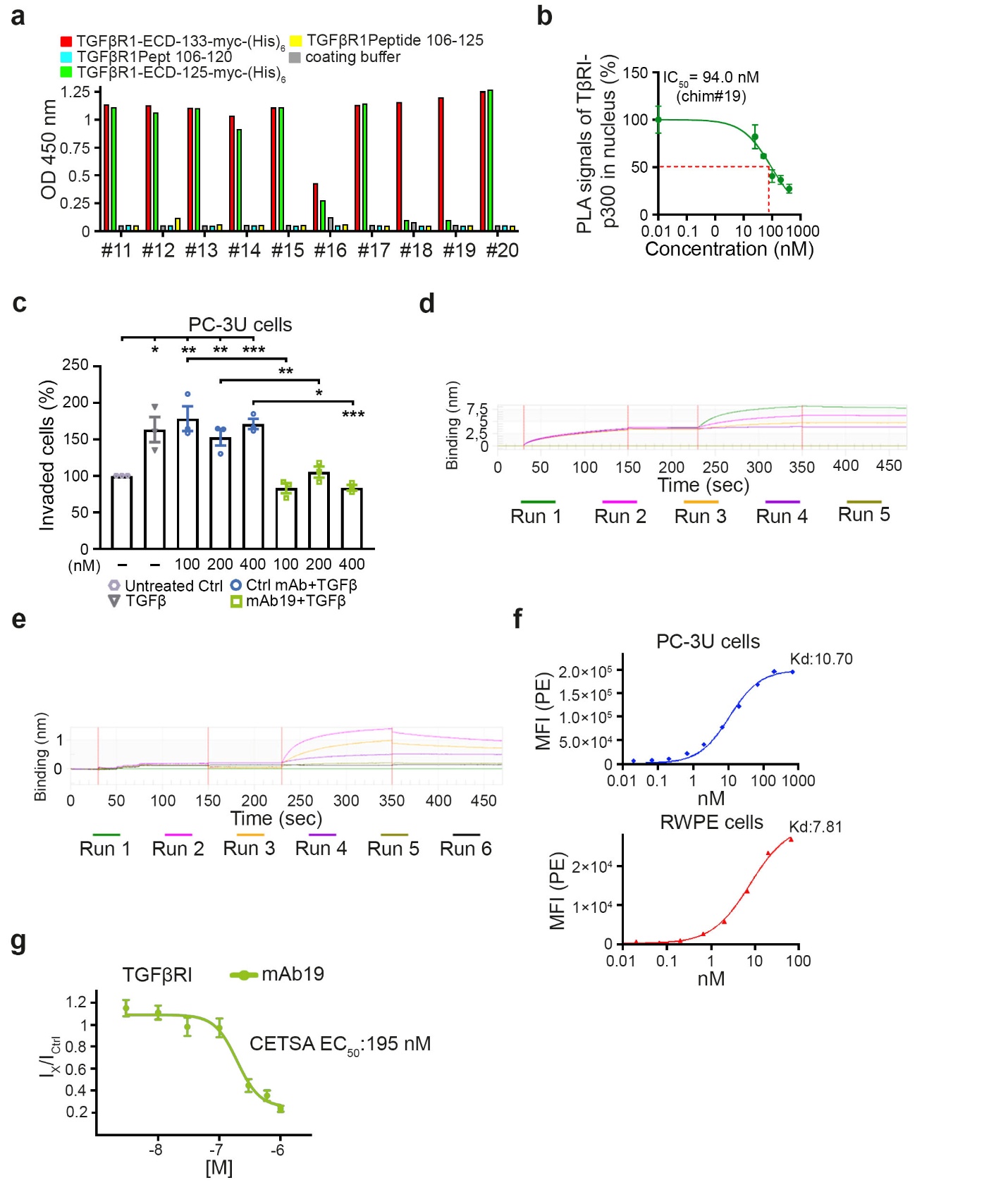


**Supplementary Figure S2.**

**Selection and characterization mAb19. a,** Periplasmatically expressed scFvs from individual clones 11-20, were screened for binding to recombinant TGFβRI-ECD-133-myc(His)_6_ and TGFβRI-ECD-125-myc(His)_6_, as well as to TβRI-derived peptides spanning amino acid residues 106-120 and 106-125. **b,** Titrated concentrations of mAb19 were incubated with TβRI-HA-reconstituted A9/PC-3U cells for 60 minutes, followed by stimulation with TGFβ1 (10 ng/ml) for 6h. The interaction between TβRI intracellular domain (TβRI-ICD) and p300 in the nucleus was subsequently assessed by *in situ* proximity ligation assay (PLA) using antibodies against TβRI-ICD-HA and p300. **c,** Treatment with mAb19 significantly prevents TGFβ-induced invasion of PC-3U cells. TGFβ1 stimulated PC-3U cells were treated with indicated concentrations of mAb19, and the cell free area bottom side of the transwell filter was quantified using ImageJ script under brightfield microscopy. Mean values ± SEMs were calculated from three independent experiments. PC-3U cells treated with TGFβ1 alone, with isotype-matched IgG1 (IgG) or left-untreated (CTRL), served as controls. Statistical significance was determined by Student’s t-test: *P < 0.05; **P < 0.01; ***P < 0.001. **d**, The dissociation constant (Kd) of mAb19 for binding to recombinant human TβRI-ECD133-myc-(His)₆ was determined using Bio-Layer Interferometry (BLI; Blitz, Sartorius). mAb19 was loaded at 200 nM (Run 1), 100 nM (Run 2), 50 nM (Run 3), and 20 nM (Run 4). As a negative control, mAb19 at 100 nM was applied in the absence of immobilized TβRI-ECD133-myc-(His)₆ (Run 5). **e**, The Kd of mAb19 for binding to human TβRI-133-huFc was determined similarly. mAb19 was loaded at 200 nM (Run 2), 100 nM (Run 3), 50 nM (Run 4), and 25 nM (Run 5). As a control, mAb 4-4-20, specific for fluorescein, was loaded at 200 nM (Run 1). Run 6 with the murine control, mAb 4-4-20 at 400 nM, was not included in the calculation. **f**, Binding of mAb19 was assessed by fluorescence-activated cell sorting (FACS) on PC-3U and RWPE-1 cells. PE geometric mean fluorescence intensity (MFI; linear scale) is plotted against the corresponding concentrations of mAb19 (log scale). **g**, CETSAR classic assays for TβRI in human prostate cancer tissue extracts were established. Target engagement by mAb19 (labeled as Tab#19 in this figure) was confirmed, and the antibody induced a small but reproducible thermal shift in the clinical material. mAb19 produced a stable and reproducible concentration–response curve, with an observed EC₅₀ of 195 nM.

Supplementary Figure S3.

**
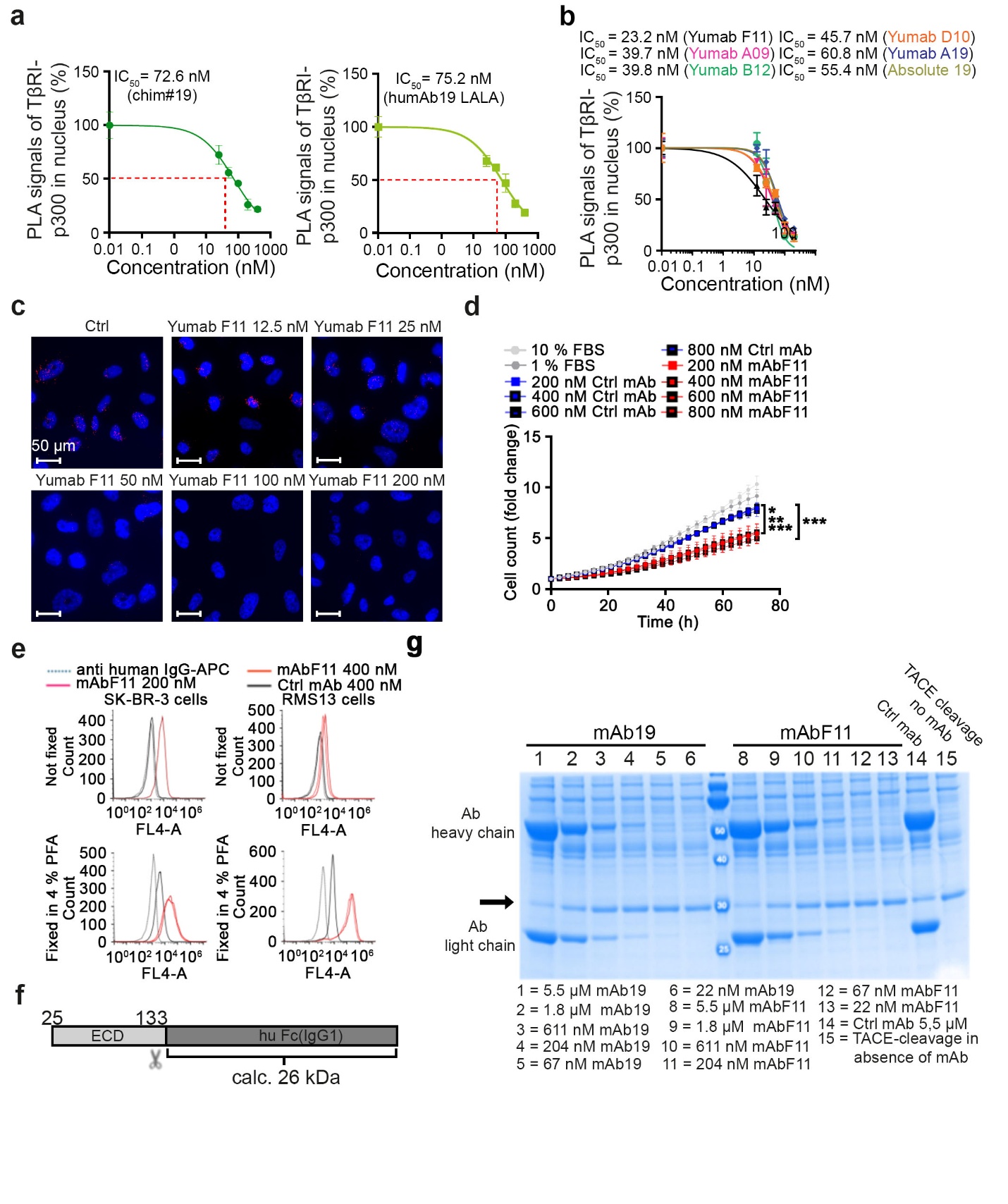
**

**Supplementary Figure S3**

**IC50 comparison of the chimeric and human versions of F11 and screening assays for affinity-matured mAb F11**

**a**, Comparison of the chimeric mouse and human version of mAb19 in inhibiting nuclear TβRI-ICD–p300 complex formation. TβRI-HA–reconstituted A9/PC-3U cells were incubated with different concentrations of mAb19 for 60 min, followed by stimulation with TGFβ for 6 h. The interaction between TβRI-ICD and p300 in the nucleus was then assessed by in situ proximity ligation assay (PLA) using antibodies against TβRI-ICD-HA and p300. **b**, Selected monoclonal antibody candidates were screened using an *in situ* proximity ligation assay (PLA) for their ability to inhibit nuclear TβRI-ICD–p300 interactions in TβRI-HA–reconstituted A9/PC-3U cells. **c**, Inhibition of nuclear TβRI-ICD–p300 complex formation by mAbF11 at the indicated concentrations, assessed using an *in situ* proximity ligation assay (PLA) in TβRI-HA–reconstituted A9/PC-3U cells. Nuclei were stained with DAPI (blue), and red dots indicate the presence of TβRI-ICD–p300 complexes. **d,** PC-3U cells were treated with 200-800 nM Ctrl mAb or 200-800 nM mAbF11 in 1 % FBS for 72 h. PC-3U cells grown in 10 % or 1 % FBS were used as controls. Growth curve was determined by using IncuCyte SX5 (Sartorius, UK). The data are presented as mean values ± SEMs from three independent experiments. Statistical significance was determined by Student’s t-test: * P<0.05, ** P< 0.01; *** P <0.001. **e**, FACS-assay to evaluate binding of mAbF11 to endogenous TGFβRI on the indicated cancer cell lines, either fixed or unfixed in 4% paraformaldehyde (PFA). **f, mAb19 and mAbF11 inhibit TACE-mediated cleavage of recombinant TGFβRI.** Schematic of recombinant TGFβRI-ECD133-huFc, comprising the extracellular domain of TGFβRI up to amino acid 133 fused to the Fc domain of human IgG1. **g,** competition assay showing inhibition of TACE-mediated cleavage of recombinant TGFβRI-ECD133-huFc in the presence of mAb19 or mAbF11 preincubated at the indicated concentrations. The cleaved Fc domain (arrow) is increasingly formed as the amount of mAb19 or mAbF11 decreases.

**Supplementary Figure S4**


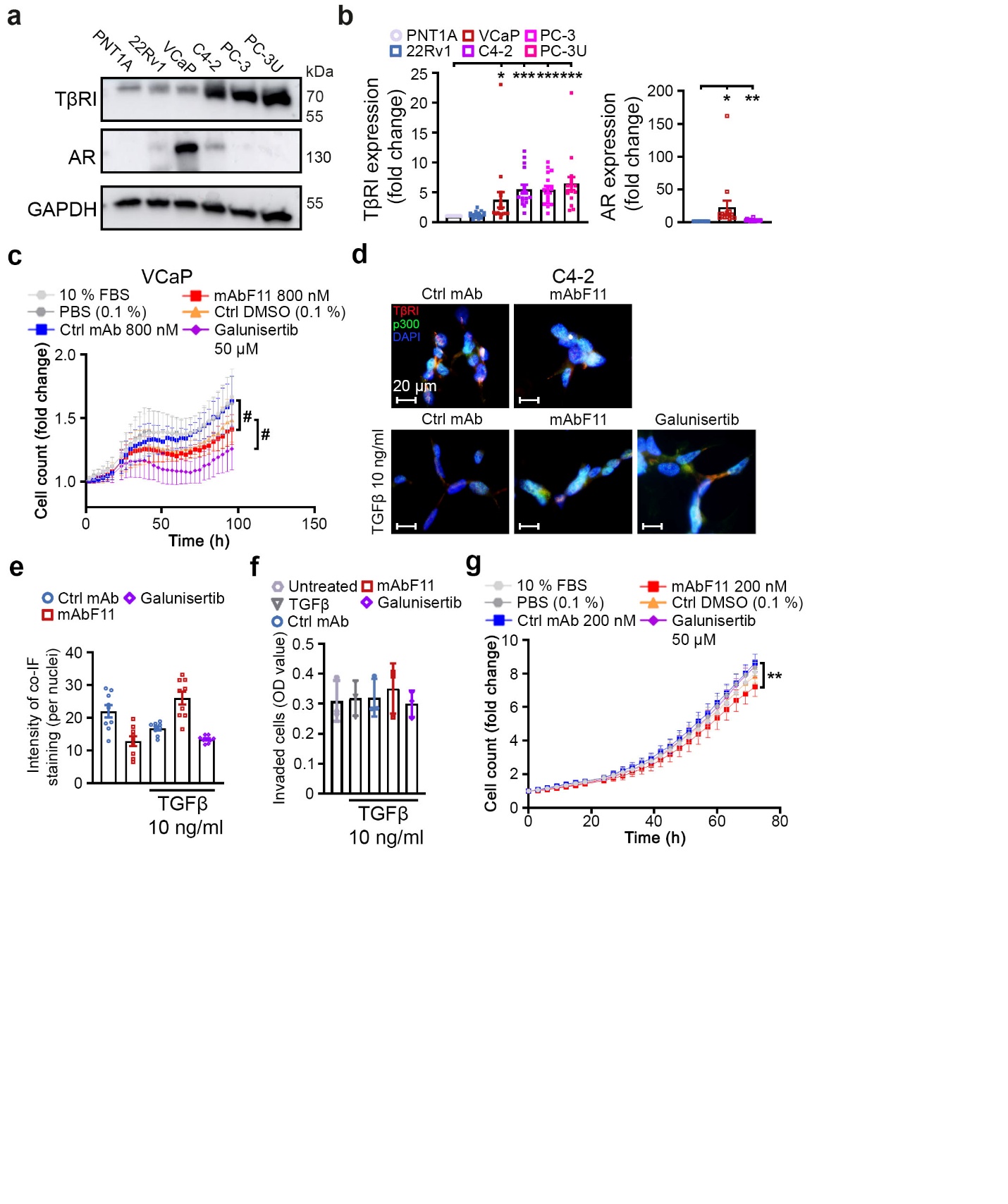


**Supplementary Figure S4**

**Characteristics of androgen-sensitive prostate cancer cells and their response to treatment with monoclonal antibodies, TGFβ and Galunisertib**

**a** Cell lysate derived from indicated human prostate epithelial and prostate cancer cells were subjected to immunoblotting with antibodies against TβRI and androgen receptor (AR). GAPDH served as internal control for equal loading of proteins. Images from a representative immunoblot experiment. **b** The intensity of immunoblots in panel a, was measured and the ratio of TβRI and AR are depicted as normalized signal of GAPDH. The data are presented as mean values ± SEMs from 17 replicates for TβRI, and 15 replicates for AR. Statistical significance was determined by Student’s t-test: * P< 0.05; ** P< 0.01; *** P <0.001. **c** VCaP cells were treated with 800 nM Ctrl mAb or 800 nM mAbF11 in 10 % FBS for 96 h. VCaP cells grown in 10 % FBS or treated with 0.1% DMSO, or galunisertib 50 μM was used for comparison cells. Growth curve was determined by using IncuCyte SX5 (Sartorius, UK). The data are presented as mean values ± SEMs from three independent experiments. Statistical significance was determined by Student’s t-test: #P<0.08. **d** Co-immunofluorescence (Co-IF) staining of TβRI (red) and p300 (green) in androgen-sensitive C4-2 cells treated as indicated for 48 h. Nuclei were stained with DAPI (blue). Scale bar, 20 μm. **e** Quantification was performed using GraphPad Prism software (version 9.4.1) Mean values ± SEMs were calculated from three independent experiments (N=3). No significant effects of stimulation with TGFβ1 or treatments were observed. **f** Invasion assay in C4-2 cells: treated or not with TGFβ for 24h with 200 nM Ctrl mAb or 200 mAbF11, or vehicle control for galunisiertib (0.1% DMSO) or galunisertib 10 μM. The optical density (OD) for C4-2 cells was quantified. Mean values ± SEMs, (N=3). No significant effect of treatments was observed. **g** C4-2 cells were treated with 200 nM Ctrl mAb or 200 nM mAbF11 in 10 % FBS for 72 h. Cells grown in 10 % FBS and treated with PBS (0.1% PBS vehicle for mAb), or 0.1% DMSO (Ctrl 0.1%), or galunisertib 50 μM was used for comparison. Growth curve was determined by using IncuCyte SX5 (Sartorius, UK). The data are presented as mean values ± SEMs from three independent experiments. Statistical significance was determined by Student’s t-test: ** P< 0.01.

**Supplementary Figure S5**

**
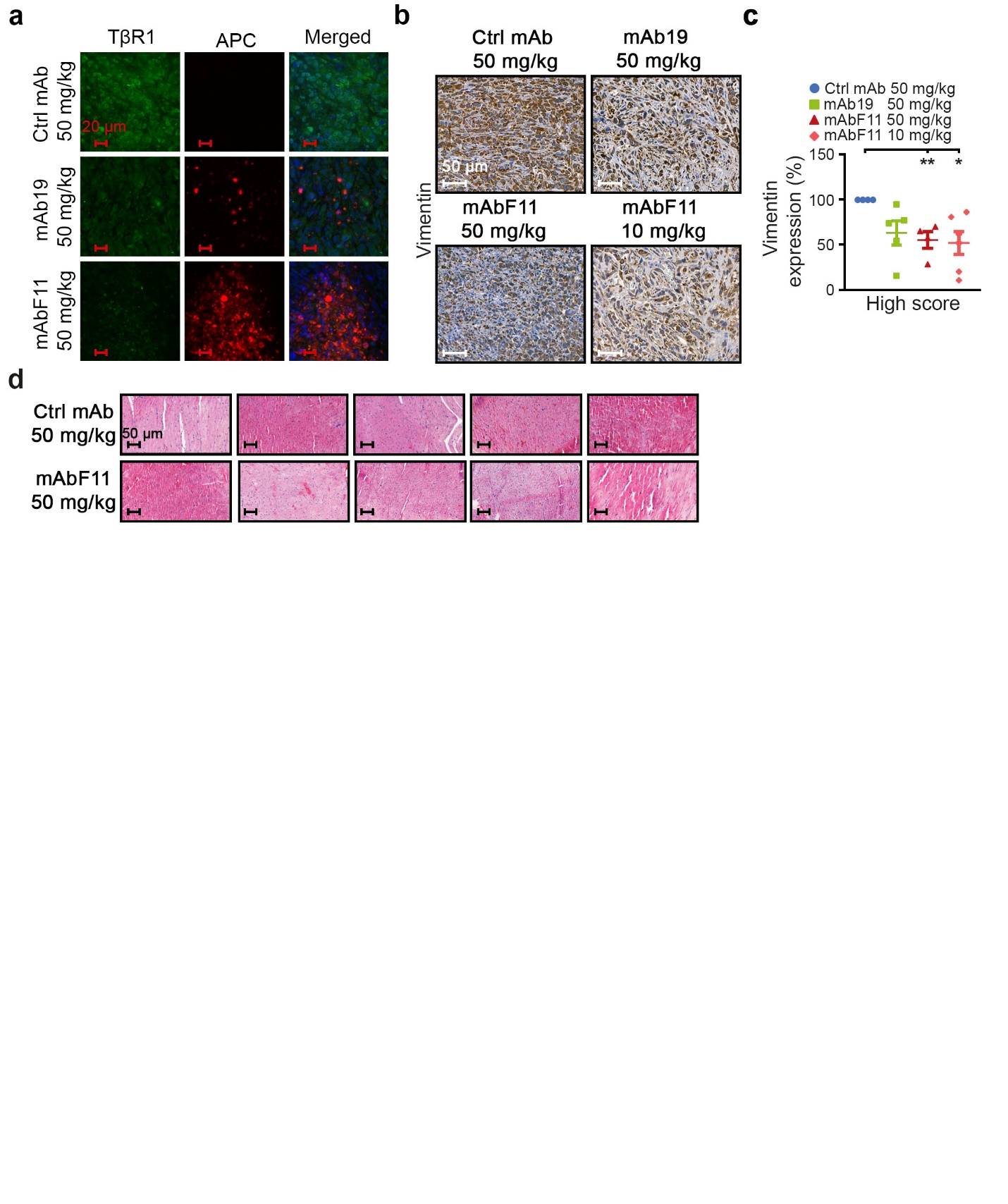
**

**Supplementary Figure S5.**

**Uptake of mAbF11 and effects of treatment on vimentin expression, cardiomyocytes, and TGFβ-induced invasion in human breast carcinoma cells.**

**a,** Tumor slides from mice treated with either Ctrl mAb 50 mg/kg, mAb19 50 mg/kg or mAbF11 50 mg/kg stained with TGFβR1 (anti-rabbit, green), APC (anti-human, red) and Hoechst (blue). Scale bar 20 μm. **b,c,** Vimentin expression after treatment with 50 mg/kg Ctrl mAb, 50 mg/kg mAb19, 50 mg/kg mAbF11 and 10 mg/kg mAbF11. Quantification of immunohistochemical staining’s was performed by using the software QuPath version 0.4.3; low (threshold 0.2 to 0.4), medium (threshold 0.4 - 0.6) and high (threshold > 0.6) staining scores are defined by intensity thresholds after cell detection. **d,** Morphology of heart tissue stained with hematoxylin and eosin after treatment with 50 mg/kg Ctrl mAb or 50 mg/kg mAbF11.

**Supplementary Figure S6**


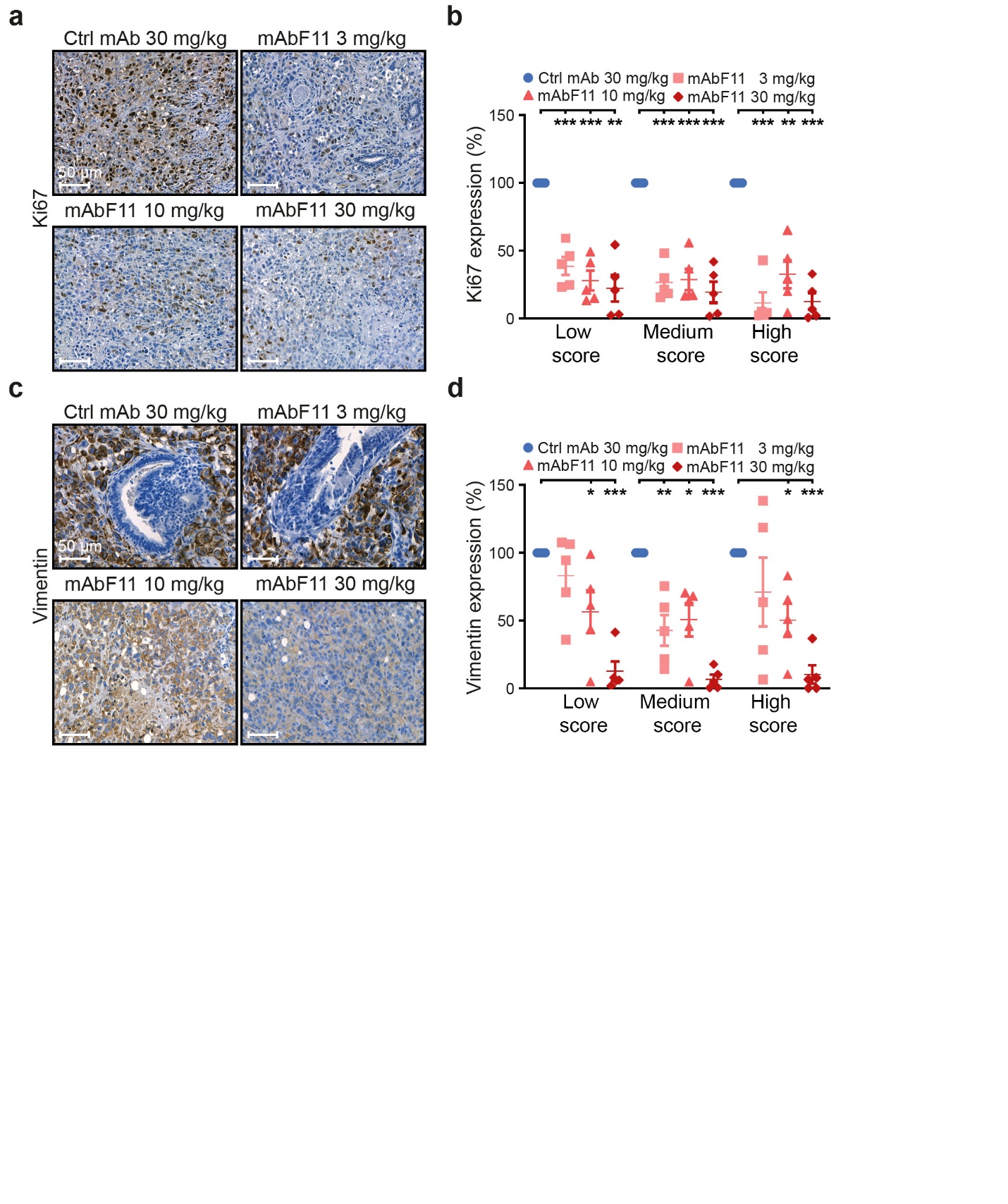


**Supplementary Figure S6.**

**Dose-dependent inhibition of TGFβ-induced proliferation and EMT upon treatment with mAb F11 in a PC-3U orthotopic xenograft model.**

**a,b,** Ki67-expression and quantification. **c,d,** Vimentin-expression and quantification. Scale bar, 50 μm. Quantification of immunohistochemical staining’s was performed by using the software QuPath version 0.4.3; low (threshold 0.2 to 0.4), medium (threshold 0.4 - 0.6) and high (threshold > 0.6) staining scores are defined by intensity thresholds after cell detection. Statistical significance was determined by Student’s t-test: * P<0.05; **P < 0.01; ***P<0.001.

Supplementary Figure S7


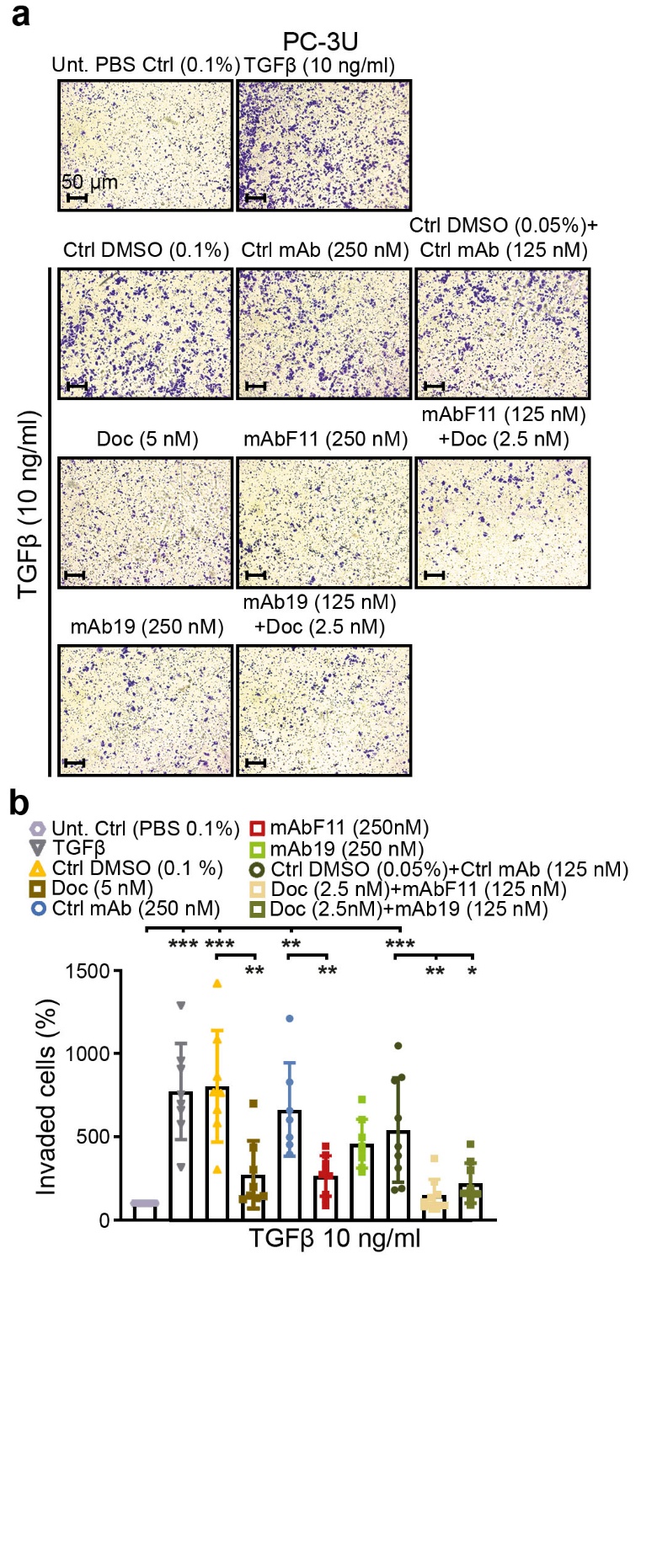


**Supplementary Figure S7.**

**Inhibition of TGFβ-induced invasion of PC-3U cells upon treatment with Docetaxel, mAb19, or mAbF11 in an *in vitro* assay**

**a,** Representative images of invaded PC-3U cells on the bottom of the transwell filter after TGFβ1 stimulation and treatment with vehicle (DMSO 0.1%), Ctrl mAb, vehicle DMSO 0.1%+ Ctrl mAb, Docetaxel, mAbF11, mAb19, mAbF11 + Docetaxel, mAb19 + Docetaxel, as indicated. Positive controls include untreated cells or cells treated with TGFβ1 alone. Scale bar, 50 μm. **b,** Quantification of invaded PC-3U cells after treatment. Raw data is presented in Supplementary Table 3. Results are shown as mean ± SEMs from three independent biological replicates, in triplicates (N=3). Statistical significance was determined by Student’s t-test: **P < 0.01; ***P < 0.001.

**Supplementary Figure S8.**


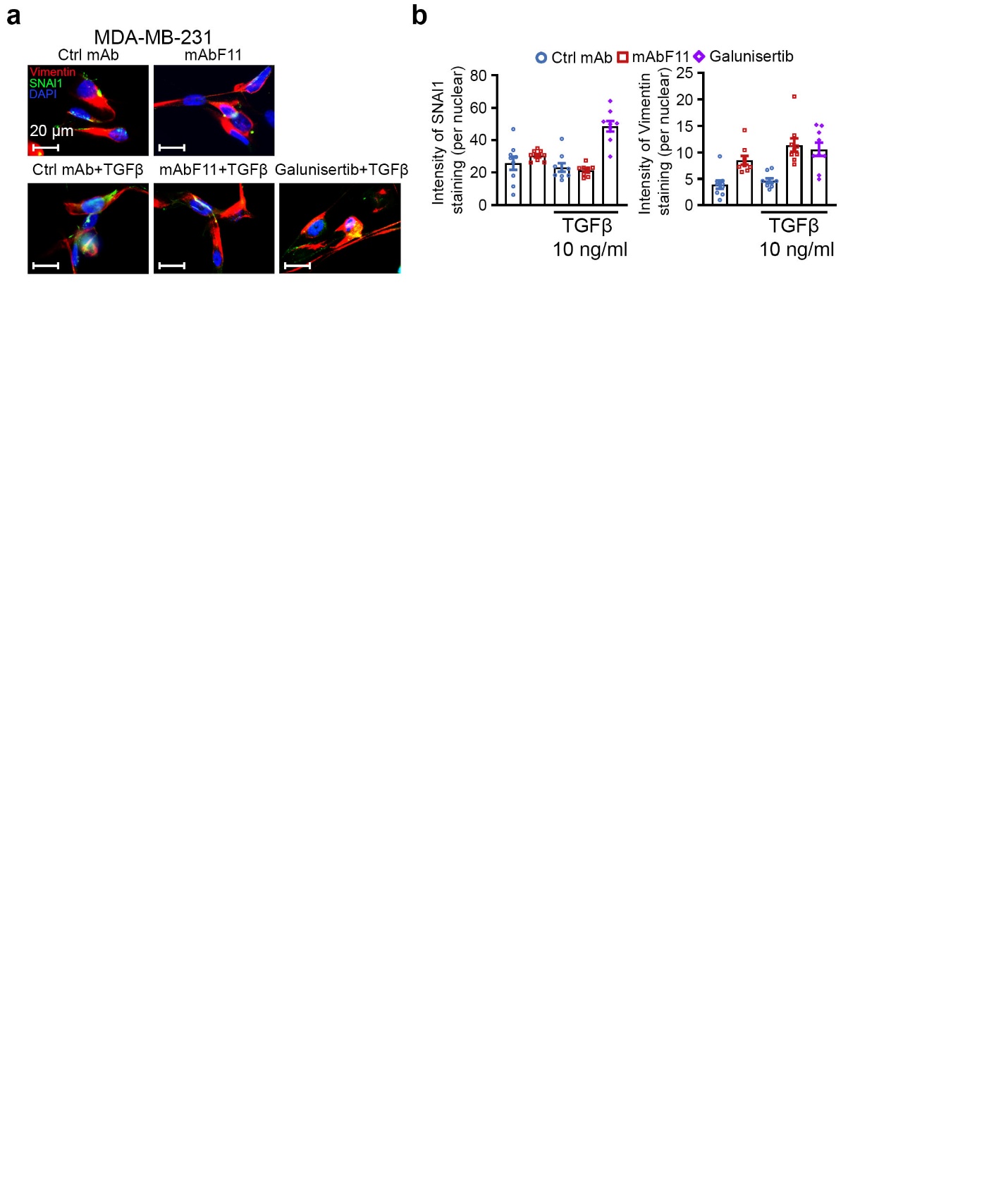


**Supplementary Figure S8.**

**Effects of stimulation of MDA-MB-231 cells with TGFβ and treatment with monoclonal antibodies; control mAb and mAbF11 on EMT markers, nuclear SNAI1 and vimentin**

**a,** co-immunofluorescence (Co-IF) staining of Vimentin (red) and SNAI1 (green) in human triple negative breast carcinoma MDA-MB-231 cells treated as indicated for 48 h. Nuclei were stained with Hoechst (blue). Scale bar, 20 μm. **b** Quantification was performed using GraphPad Prism software (version 9.4.1) Mean values ± SEMs were calculated from three independent experiments, in triplicates (N=3). No significant effects of stimulation with TGFβ1 or treatments were observed.

**Supplementary Table 1**

**Supplementary Table S1**

Kd and EC50-values of selected antibody candidates determined by Fluorescence-activated cell sorting (FACS). The best performing antibody candidates chosen for follow up studies are marked in green.

**Supplementary Table S2**

**Table S2**

| Compound | Concentration |  | In vivo/clinic | Reference |  |
| --- | --- | --- | --- | --- | --- |
|  |  |  |  |  |  |

Docetaxel 10 mg/kg In vivo ^1-3^

Docetaxel 25 mg/kg In vivo ^4^

| Compound | Concentration |  | In vivo/clinic | Reference |  |
| --- | --- | --- | --- | --- | --- |
|  |  |  |  |  |  |

Docetaxel 50-75 mg/mm^2^ Clinic ^5^

Docetaxel 30-75 mg/mm^2^ Clinic ^6^

Docetaxel 55-75 mg/mm^2^ Clinic ^7^

Docetaxel 75 mg/mm^2^ Clinic ^8^

**Supplementary Table S2.**

Current literature about concentrations of docetaxel used in preclinical studies performed *in vivo* as well as in treatment of patients with cancer.

1 Chen, L. *et al.* Circ_0004087 interaction with SND1 promotes docetaxel resistance in prostate cancer by boosting the mitosis error correction mechanism. *J Exp Clin Cancer Res* **41**, 194 (2022).

2 Yin, H. *et al.* circCYP24A1 promotes Docetaxel resistance in prostate Cancer by Upregulating ALDH1A3. *Biomark Res* **10**, 48 (2022).

3 Ma, Z. *et al.* Docetaxel remodels prostate cancer immune microenvironment and enhances checkpoint inhibitor-based immunotherapy. *Theranostics* **12**, 4965-4979 (2022).

4 Dong, X. & Zhang, J. Maximum tolerated dose and toxicity evaluation of orally administered docetaxel granule in mice. *Toxicol Rep* **12**, 430-435 (2024).

5 Martinez-Recio, S. *et al.* Comparison of 2-Weekly and 3-Weekly Dosing of Docetaxel in Metastatic Prostate Cancer. *Clin Genitourin Cancer* **20**, 363-370 (2022).

6 Zhao, J., Guercio, B. J. & Sahasrabudhe, D. Current Trends in Chemotherapy in the Treatment of Metastatic Prostate Cancer. *Cancers (Basel)* **15** (2023).

7 Sweeney CJ. *et al*. Chemohormonal therapy in metastatic hormone-sensitive prostate cancer. *N Engl J Med* **373**, 737-46 (2015).

8 Sonpavde, G. *et al.* Randomized phase II trial of docetaxel plus prednisone in combination with placebo or AT-101, an oral small molecule Bcl-2 family antagonist, as first-line therapy for metastatic castration-resistant prostate cancer. *Ann Oncol* **23**, 1803-1808 (2012).

**Supplementary Table 3**

**Supplementary Table S3**

Quantitative analyses of number of PC-3U cells in invasion assay treated as indicated in Supplementary Figure 7 a,b.

**Supplementary Table S4**

| **Antibody** | **Cat number** | **Work concentration** | **Supplier** |
| --- | --- | --- | --- |
|  |  |  |  |
| **Primary antibodies for Western blot** | | | |
| pSMAD2 | 3108S | 1:1000 | Cell Signaling |
| SMAD2 | 3103S | 1:1000 | Cell Signaling |
| pp38 | 9211S | 1:1000 | Cell Signaling |
| p38 | 9217S | 1:1000 | Cell Signaling |
| pAKT | 4060S | 1:1000 | Cell Signaling |
| AKT | 2920S | 1:1000 | Cell Signaling |
| pp65 | 3033S | 1:1000 | Cell Signaling |
| p65 | 8242S | 1:1000 | Cell Signaling |
| p300 | AF3789 | 1:100-1000 | R&D |
| TGFβRI | 235578 | 1:250-1000 | Abcam |
| anti-TGFBR1 antibody | ab31013 | 1:1000 | Abcam |
| Androgen Receptor | MA5-13426 | 1:1000 | Thermo Fisher Scientific |
| GAPDH | 60004-1-Ig | 1:80 000 | Proteintech |
| Actin | A5441 | 1:5000 | Sigma |
| **Primary antibodies for immunostaining** | | | |
| SNAI 1 Antibody (T-18) | sc-10433 | 1:100 | Santa Cruz |
| Vimentin (D21H3) | #5741 | 1:100 | Cell Signaling |
| TGFβRI (V-22) | sc-398 | 1:100 | Santa Cruz |
| p300 | AF3789 | 1:100 | R&D |
| TGFβRI | PA5-98192 | 1:200 | Thermo Fisher Scientific |
| **Primary antibodies for immunohistochemistry** | | | |
| TGFβRI | PA5-98192 | 1:500 | Thermo Fisher Scientific |
| Ki67 | ab92742 | 1:250 | Abcam |
| Vimentin | MA5-16409 | 1:100 | Thermo Fisher Scientific |
| anti-Vimentin | ab16700 | 1:200 | Abcam |
| **Primary antibodies for immunoprecipitation** | | | |
| TβRI | 235578 | 1:1000 | Abcam |
| p300 | sc-584 | 1:1000 | Santa Cruz |
| p300 | sc-48343 | 1:1000 | Santa Cruz |
| **Primary antibodies for PLA** | | | |
| HA | 3724S | 1:200 | Cell Signaling |
| p300 | AF3789 | 1:100- 1:200 | R&D Systems |
| TGFβRI | PA5-98192 | 1:500 | Thermo Fisher Scientific |
| **Secondary antibodies for Western blot** | | | |
| anti rabbit immunoglobulins/ HRP | P0448 | 1:10000 | Agilent Technologies |
| anti mouse immunoglobulins/ HRP | P044701-2 | 1:15000 | Agilent Technologies |
| **Secondary antibodies for immunostaining** | | | |
| donkey-anti-goat Alexa488 | 705-546-147 | 1:300 | Jackson ImmunoResearch |
| donkey-anti-Rabbit Cy™3 | 711-166-152 | 1:300 | Jackson ImmunoResearch |
| anti rabbit Alexa fluor 488 | A3271 | 1:500 | Thermo Fisher Scientific |
| anti human APC | 709-136-149 | 1:200 | Novakemi |
| **Secondary antibodies for immunohistochemistry** | | | |
| HRP-conjugated secondary antibody | K4003 |  | Agilent Technologies |
| Bright Vision HRP | nr DPVR110HRP |  | Immunologic |
| **Secondary antibodies for immunoprecipitation** | | | |
| anti rabbit light chain specific | 211-032-171 |  | Jackson ImmunoResearch |

**Supplementary Table S4.**

Information about antibodies used in experiments described in main article and the dilutions of antibodies.

**Supplementary Table S5**

| Primers used in qRT-PCR | |
| --- | --- |
| Gene | Primers(5'- 3') |
| *ZEB1* [Homo sapiens (human)] | F: CAGCTTGATACCTGTGAATGGG |
|  | R: TATCTGTGGTCGTGTGGGACT |
| *TGFBR1* [Homo sapiens (human)] | F: GCTGTATTGCAGACTTAGGACTG |
|  | R: TTTTTGTTCCCACTCTGTGGTT |
| *ALDH1A3*  [Homo sapiens (human)] | F: CCCTGGAGACGATGGATACAG |
|  | R: TCTGAGGGTTCTAATACAGCCC |
| *KLK3* [Homo sapiens (human)] | F: GTGTGTGGACCTCCATGTTATT |
|  | R: CCACTCACCTTTCCCCTCAAG |
| *NKX3-1* [Homo sapiens (human)] | F: CCCACACTCAGGTGATCGAG |
|  | R: GAGCTGCTTTCGCTTAGTCTT |
| *SNAI 1* [Homo sapiens (human)] | F: CCTTCTCTAGGCCCTGGCT |
|  | R: AGGTTGGAGCGGTCAGC |
| *AR* [Homo sapiens (human)] | F: CCAGGGACCATGTTTTGCC |
|  | R : CGAAGACGACAAGATGGACAA |
| *AR* [Homo sapiens (human)] | F: GACGACCAGATGGCTGTCATT |
|  | R: GGGCGAAGTAGAGCATCCT |
| *EP 300* [Homo sapiens (human)] | F: TTCCCCTAACCTCAATATGGGAG |
|  | R: GCCTGTGTCATTGGGCTTTTG |
| *HGF* [Homo sapiens (human)] | F: GCTATCGGGGTAAAGACCTACA |
|  | R: CGTAGCGTACCTCTGGATTGC |
| *MYC* [Homo sapiens (human)] | F: TCCCTCCACTCGGAAGGAC |
|  | R: CTGGTGCATTTTCGGTTGTTG |
| *VIM [Homo sapiens (human)]* | F: GCTCGTCACCTTCGTGAATA |
|  | R: AGTTTGGAAGAGGCAGAGA |
| *GAPDH*  [Homo sapiens (human)] | F: AAATCCCAT CAC CATCTTCC |
|  | R: AAATGAGCCCCAGCCTTC |

**Supplementary Table S5**

Primer sequences used for RT-qPCR related to Figure 4 e,f and Figure 5 f,g.
